# Addressing global hotspots of drought-related crop production losses

**DOI:** 10.1038/s41467-026-72715-y

**Published:** 2026-05-19

**Authors:** Marta Tuninetti, Kyle Frankel Davis

**Affiliations:** 1 Department of Environment, Land, and Infrastructure Engineering, Politecnico di Torino, Turin, Italy; 2 Department of Geography and Spatial Sciences, University of Delaware, Newark, DE USA; 3 Department of Plant and Soil Sciences, University of Delaware, Newark, DE USA; 4 Data Science Institute, University of Delaware, Newark, DE USA

**Keywords:** Hydrology, Environmental sciences, Agroecology

## Abstract

Meeting future food demand requires transforming food systems to simultaneously increase production, reduce environmental impacts, and adapt to climate change. As climate variability increasingly affects production stability, understanding where cropping patterns are vulnerable to hydroclimatic stress is a missing but critical step toward improving agricultural production resilience. Here we combine gridded climate data, spatially explicit agricultural statistics, and empirical water-production functions to quantify global patterns of rainfed and irrigated crop-specific Drought Sensitivity-defined as the percent reduction in median yield under extreme hydroclimatic conditions—and drought-associated losses for 17 major crops, representing 75% of global production. This metric identifies locations where crops experience high climate variability and are most susceptible to drought-related losses. We estimate global losses of  − 10.1% and  − 6.8% in median rainfed and irrigated production, respectively, under historically observed extremes—enough calories to feed 2.1 billion people—and identify hotspots in the central US, eastern Brazil, the Mediterranean, and South Asia. Focusing on monsoon cereals (rice, maize, millet, sorghum), we show that sustainable irrigation expansion and targeted crop switching could avoid 62% of rainfed losses while increasing median production by 14%. This scalable framework enables proactive targeting of mitigation actions and investments to stabilize and increase global crop supply.

## Abstract (web summary)

Climate extremes increasingly threaten global crop production stability. This study quantifies drought sensitivity for major crops worldwide, identifies production hotspots, and suggests that irrigation expansion and crop switching could reduce losses by 62% and increase median production by 14%.

## Introduction

Global food supply has increased markedly since the 1960s to meet the demands of a growing and more affluent population and to prevent widespread hunger and famine ^1^. This tripling of food production was made possible by a narrow set of crops (e.g., rice, maize, wheat)^2^, and as a result, staple crop production has become more homogeneous^3^—shifting away from more nutritious cereals and towards higher-yielding^4^ and less climate-resilient production mixes^5^. At the same time, climate change, climate variability, and extreme events are exerting growing pressure on crop production systems around the planet^6^. Thus, while there is a clear need to increase food supply, minimize environmental impacts and improve nutrition in the coming decades^7,8^, a critical condition for achieving more sustainable food production is ensuring its stability under varying climatic conditions^9,10^. The variability of crop production from year to year depends in large part on the sensitivity of crop yields to variations in climate^11–13^—a relationship with profound implications for food supply and rural livelihoods. Recent work has shown that globally, more than a third of yield variability in major crops can be explained by climate variations ^11^ and climate extremes ^14^. Numerous other country-focused studies have further shown the influence of seasonal temperature and rainfall (e.g., refs. ^15–19^) and extreme events (e.g., droughts, heatwaves, floods) on yield variability. All of this research points to differing levels of climate sensitivity between crops and between regions. With climate variability expected to increase throughout the century^20^, measures to buffer crop production against these stresses are a critical aspect of climate adaptation. Yet, despite the importance of enhancing the climate resilience of food production, limited work has investigated global patterns of crop yield responses to climate variability, focusing only on a few major crops (i.e., maize, rice, soybean, wheat) or coarse spatial scales (i.e., sub-national) and relying largely on statistical approaches that incorporate historical agricultural statistics^11,14,21^. For drought in particular, various indices have been developed to predict short- and long-term drought conditions— including the Palmer Drought Index^22^, Standardized Precipitation Index^23^, Crop Moisture Index^24^, Surface Water Supply Index^25^, Vegetation Health Index^26^, and Vegetation Drought Response Index^27^—but these indices are not typically tailored to the sensitivities of specific crops.

Therefore, there remains a poor understanding of the variability of yields due to climate variations (and drought in particular) throughout the entire crop production basket and at fine spatial granularity, limiting the extent to which climate adaptation efforts can be targeted in the most vulnerable areas and for a variety of crop mixes^28–32^. Here we fill this critical knowledge gap by quantifying and analyzing global patterns of drought sensitivity (i.e., *DS*, the responsiveness of a crop’s yield to moisture anomalies driven by climate variability) for 17 of the world’s major crops—barley, cassava, cotton, groundnut, maize, millet, oil palm, potatoes, rapeseed, rice, sorghum, soybean, sugar beet, sugarcane, sweet potatoes, wheat, and yams—which constitute 75% of global primary crop production^2^. First, we combine historical climate and soil data and global gridded maps of crop harvested area within a daily soil water balance model to estimate pixel-level (5 arcminute; 10 km at the equator) crop-specific actual evapotranspiration (*ET*_*a*_) for each growing season^33^ between 1961 and 2018. We then employ an empirical water production function that relates yield and *ET*_*a*_^34–37^ to estimate changes in current yields under historical climate conditions. From this, we calculate the pixel-level drought sensitivity of each crop—measured as the percentage difference in yield under median *ET*_*a*_ (i.e., typical climate conditions) and 10th percentile *ET*_*a*_ (i.e., observed extreme adverse climate-driven moisture conditions)—and estimate climate-related production losses under historically observed extreme climate conditions. The *DS* indicator does not directly account for the effects of temperature extremes or other non-hydrological climate stresses on crop yield; rather, it captures their influence only insofar as they alter water availability and evapotranspiration, which in turn affect yield. Finally, we evaluate opportunities to address hotspots of drought sensitivity—by maintaining or increasing production levels and reducing climate-related production losses—through sustainable irrigation expansion (i.e., only in locations with no blue (both ground and surface) water scarcity^38–41^ and selective switching to less drought-sensitive cropping choices. The approach developed here can serve as a transferable and scale-neutral method for evaluating the current state of crop production (in)stability due to climate variability at multiple spatial scales—an essential consideration when seeking to achieve greater food system resilience—and for developing agricultural climate adaptation strategies tailored to local needs.

## Results

### Global hotspots of rainfed drought sensitivity

**Fig. 1:**
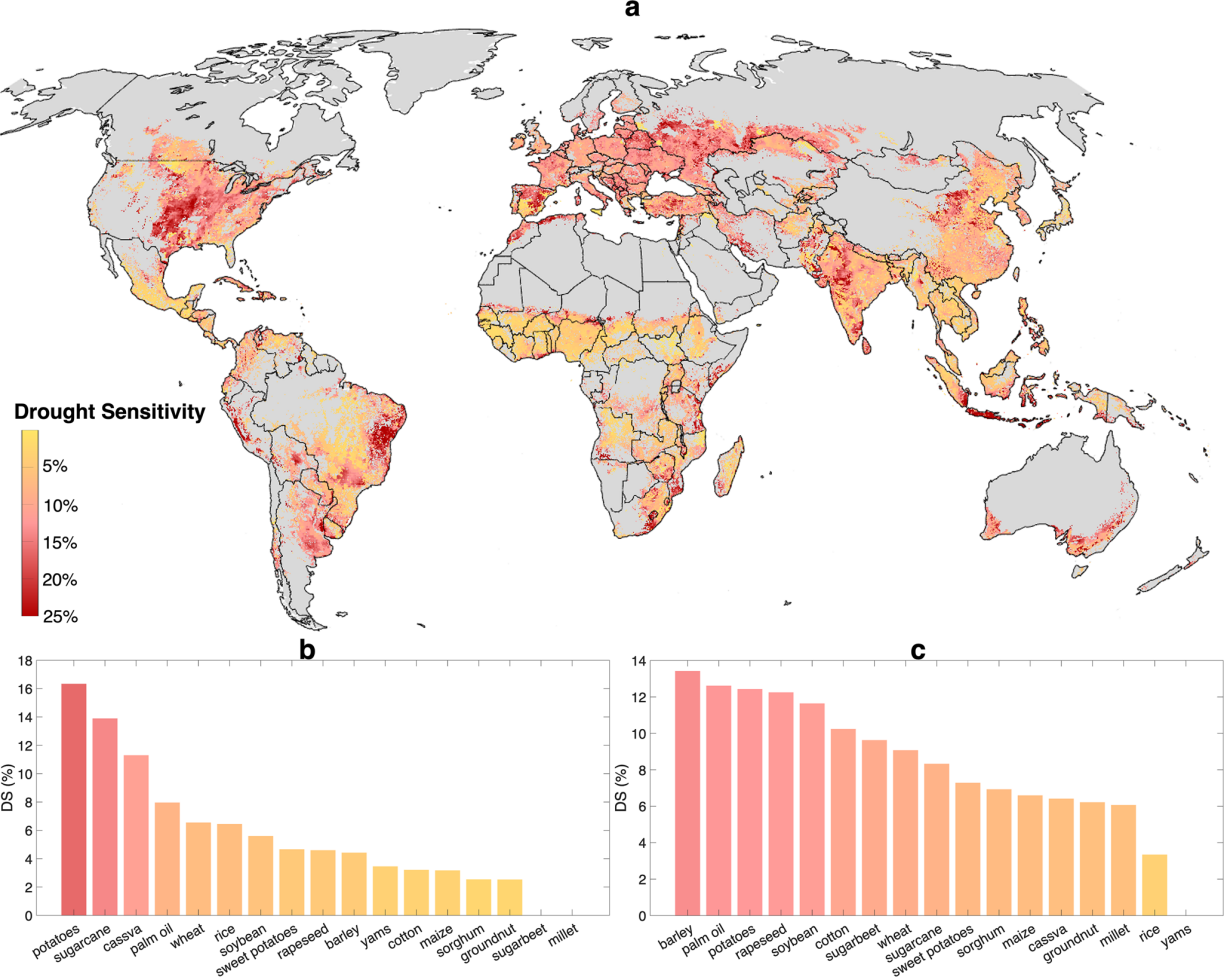
Hotspots of drought sensitivity for global rainfed crop production. Pixel-level drought sensitivity (*DS*) of total crop production over the period 1961–2018 (**a**). Country-average drought sensitivities in Brazil (**b**) and in China (**c**) are shown as illustrative examples because of their relatively high crop diversity and geographic diversity.

Crop drought sensitivity (*DS*) measures the percent of the median yield that is lost for a crop when the evapotranspiration falls to the lower 10th percentile of its distribution evaluated over the period 1961–2018. Because actual evapotranspiration (*ET*_*a*_) is jointly controlled by soil moisture availability and atmospheric evaporative demand (*ET*_0_), the lower tail of the *ET*_*a*_ distribution may reflect either water-limited (precipitation-driven) or energy-limited (radiation- and temperature-driven) growing conditions. Accordingly, our drought sensitivity indicator captures yield reductions arising from both hydrological drought and reduced atmospheric demand, depending on the dominant climatic constraint in each region and crop system. Globally, we estimate rainfed production losses (Fig. 1, Figs. S1–3 for crop-specific *DS*) due to climate variability to be  − 10.1% (and  − 6.8% for irrigated production, see Table 1). This is equal to a loss of 1.5 ⋅ 10^15^ calories under rainfed conditions (0.5 ⋅ 10^15^ calories under irrigated conditions)—enough to feed 1.6 (0.5) billion people, assuming an average per capita intake of 2500 kcal per day^42^. The highest global average *DS* values (Table 1) are found for rainfed soybean (15.2%), potatoes (13.5%), and rapeseed (12.6%), while yams (2.8%), groundnuts (6.2%), and millet (6.8%) are the least sensitive to climate variability, despite local exceptions. These relative sensitivities are in agreement with the agronomic understanding of these crops’ drought tolerance^43^. For most of the study crops, precipitation is lower than the 20th percentile in  > 90% of the cultivated pixels, confirming the critical role of rainfall scarcity in inducing moisture anomalies (water-limited crops) and, thus, yield losses (see Fig. S4). However, sorghum, millet, rapeseed, and yams exhibit different behavior as *ET*0 anomalies seem to be the most relevant drivers in influencing crop evapotranspiration reduction (energy-limited crops) and, thus, yield (see Fig. S4). Their lowest *ET*_*a*_ years occur when atmospheric evaporative demand is reduced, often due to cooler, cloudier, or less windy conditions that limit energy availability for evapotranspiration. In these cases, the constraint is not water supply (and the soil moisture availability) but reduced climatic drivers of evaporation and transpiration (atmospheric moisture), which can shorten growth duration or limit photosynthesis, ultimately reducing crop water use and productivity.

**Table 1:** Summary of global current production and expected production losses due to extreme climatic conditions

	*P*_*rf*_ [Mton]	*P*_*ir*_ [Mton]	*L*_*rf*_ [Mton]	*L*_*ir*_ [Mton]	*P*_*ir*_ [% of total]	*L*_*rf*_ [%]	*L*_*ir*_ [%]
barley	121	15	15	2	10.8	12.5	10.7
cassava	238	5	26	0	2.2	11.1	0.1
cotton	26	44	2	2	62.7	9.2	4.8
groundnuts	30	10	2	0	25.0	6.2	4.9
maize	640	213	55	11	25.0	8.5	5.3
millet	22	2	1	0	7.8	6.8	4.6
palm oil	229	0	19	0	0.2	8.3	0.4
potatoes	266	80	36	7	23.1	13.5	8.8
rapeseed	56	6	7	0	9.9	12.6	2.5
rice	159	544	11	41	77.4	7.1	7.5
sorghum	48	10	3	1	17.9	7.1	5.3
soybean	237	13	36	1	5.3	15.2	9.6
sugarbeet	178	67	20	5	27.5	11.5	7.3
sugarcane	1001	719	119	45	41.8	11.9	6.2
sweet potatoes	101	3	7	0	3.1	7.2	7.8
wheat	432	243	43	20	36.0	9.9	8.1
yams	53	0	1	0	0.0	2.8	4.9
all crops	3838	1976	406	135	34.0	10.6	6.8

Current (circa year 2010) global production under rainfed (*P*_*rf*_) and irrigated (*P*_*ir*_) conditions was estimated from the MAPSPAM dataset^89^. Expected rainfed (*L*_*rf*_) and irrigated (*L*_*ir*_) production losses under extreme climatic conditions were estimated as the product of *CS*, yield, and harvested area.

Examining the pixel-level average drought sensitivity for all the study rainfed crops (Fig. 1), we find that critical *DS* hotspots are unevenly distributed worldwide and are associated with different crops (Fig. S3), with many hotspots falling in breadbasket regions (Fig. 1 and S1–2). In the United States, we find high drought sensitivity (soybean, barley, and sorghum) in the Midwest, where freshwater resources are already stressed beyond sustainable rates^44–46^. Brazil shows high sensitivity in the Eastern Caatinga and across the Mata Atlantica biomes (potatoes, sugarcane, cassava, Fig. 1b) while China exhibits larger *DS* for barley, palm oil and potatoes. Notably, rice production in China shows the lowest *DS* among the study crops. The Mediterranean basin also presents critical hotspots of *DS,* especially in Spain (rapeseed and sweet potatoes), Morocco (potatoes and barley), and Algeria (wheat and barley) (see Supplementary Data 1 and 2). Crop production in sub-Saharan Africa is moderately sensitive to climate variability in the Sahel region and in East African countries.

Overall, country-averaged rainfed *DS*s show significant positive correlation with national rainfed production (expressed in tonnes) for 10 out of 17 crops (Fig. S5), suggesting that major producing areas also tend to have greater sensitivity to climate variability.

### Sustainable irrigation expansion to avoid climate-related production losses

**Fig. 2:**
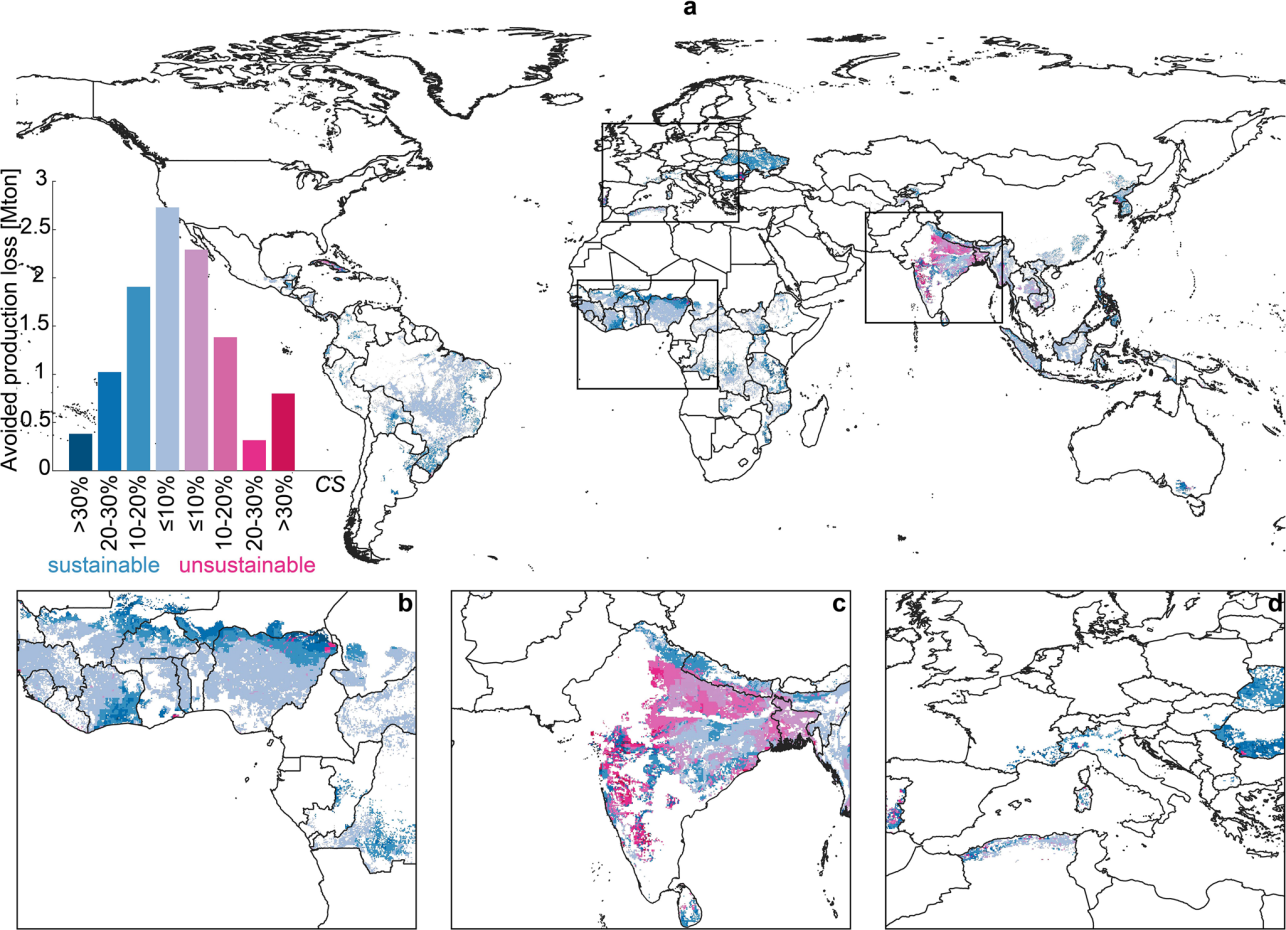
Potential for reducing rice's drought sensitivity through irrigation expansion. The map shows the drought sensitivity *DS* of rice production and the potential for reducing it through irrigation expansion (**a**). Pink cells highlight the most critical hotspots of *DS* where sustainable irrigation expansion is not possible. Blue cells indicate areas where irrigation can be sustainably expanded as a strategy to reduce *DS*. The inset histogram shows the expected avoided production loss [million tons] due to irrigation expansion. Panels are provided for several critical rice-producing areas: West Africa (**b**), India (**c**), and the Mediterranean basin (**d**).

With two-thirds of cultivated areas currently rainfed and because irrigation is known to provide benefits in reducing the effects of climate extremes on crop productivity, we then assessed the potential to sustainably expand irrigation (see Methods) into currently rainfed areas (i.e., in locations that do not currently experience physical (ground and surface) water scarcity^41^), in order to reduce drought-related production losses. Because they fall within the same crop category and play a similar role in diets, we focus the remainder of the results on the example of monsoon cereals (i.e., maize, millet, rice, and sorghum), noting that this analysis can also be extended to investigate potential switching between other crops depending on the cropping systems within a specific location (see Methods). We find that rainfed production losses could be reduced by 60% thanks to sustainable irrigation expansion, while allowing a global median production gain of 12%. Many of the regions that would benefit most from this expansion are also places facing persistent nutrition insecurity, including Nigeria, South Sudan, and Venezuela^47^. Taking the example of rice (Fig. 2), we see that many locations show great potential for the sustainable expansion of irrigation to reduce drought sensitivity—especially in Sub-Saharan Africa (Fig. 2b). However, critical hotspots of high *DS* also emerge in locations where irrigation cannot be sustainably expanded—for instance, across Bangladesh and India (Fig. 2c), where rice is a key dietary staple that provides (together with wheat) up to 50% of total food energy and protein^48^. This example using rice demonstrates the utility of our findings in providing high-resolution outputs for identifying where opportunities exist globally for irrigation expansion to address drought vulnerability for a variety of major crops (see also Figs. S6–9), information which is key to aligning climate adaptation and food security goals.

### Opportunities for crop switching

**Fig. 3:**
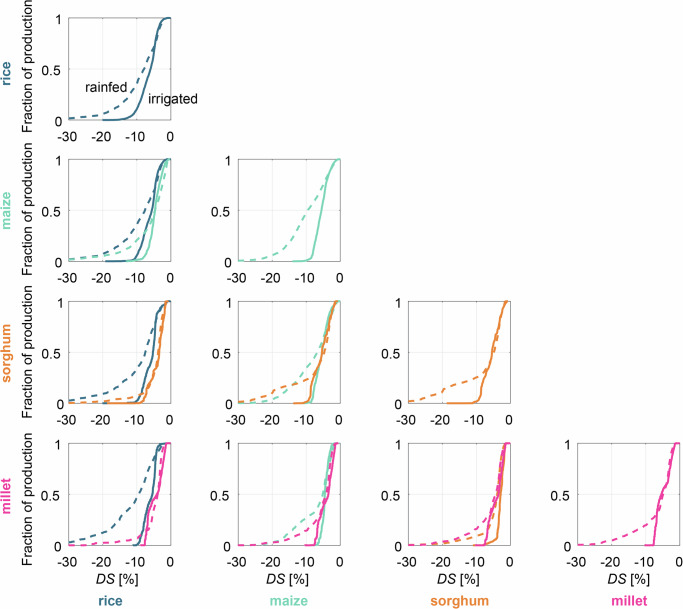
Paired comparisons of cumulative drought sensitivity. The main diagonal of the figure shows production-weighted CDFs (normalized by total production; *y*-axis) of the crop-specific drought sensitivity (*DS*) for rainfed (dashed line) and irrigated (solid line) crops. All charts below the diagonal show the CDFs of the *DS* associated with pairs of crops, considering only those pixels where both crops co-occur. CDFs shifted towards the right of each plot indicate that a larger fraction of that crop’s production is subject to lower levels of drought sensitivity. For instance, rainfed maize is less drought-sensitive than rainfed rice.

We also examined opportunities for crop switching (see Methods) as an adaptation strategy to climate variability in the areas where water scarcity constrains irrigation expansion (e.g., Fig. 2 for rice). By examining the production-weighted cumulative distribution function (CDF) of *DS* among pairs of monsoon crops (i.e., only in those pixels where both crops are cultivated), it is possible to directly compare the *DS*s of two crops and to determine which of the two crops will tend to experience smaller yield variability and production losses under extreme climate conditions (Fig. 3). In doing so, we find that maize, millet, and sorghum all clearly outperform irrigated and rainfed rice but that the relative drought sensitivity performance of the other paired comparisons (i.e., maize-millet, maize-sorghum, millet-sorghum) is more nuanced and location-dependent (Fig. 3 and Fig. S6). As such, a pixel-by-pixel assessment (performed in the next section) is necessary to determine which crops may provide reductions in *DS* relative to current cropping patterns (see Figs. S6–9). We note that the calculations of these CDFs are spatially flexible and can be adapted to investigate the relative drought sensitivity of crops within specific world regions, countries, or sub-national areas of interest. Such outcomes can provide invaluable information for decision-making at multiple spatial scales regarding the promotion of drought-resilient crops in the most appropriate and climatically suitable locations.

### Integrated solutions to buffer crop production

**Fig. 4:**
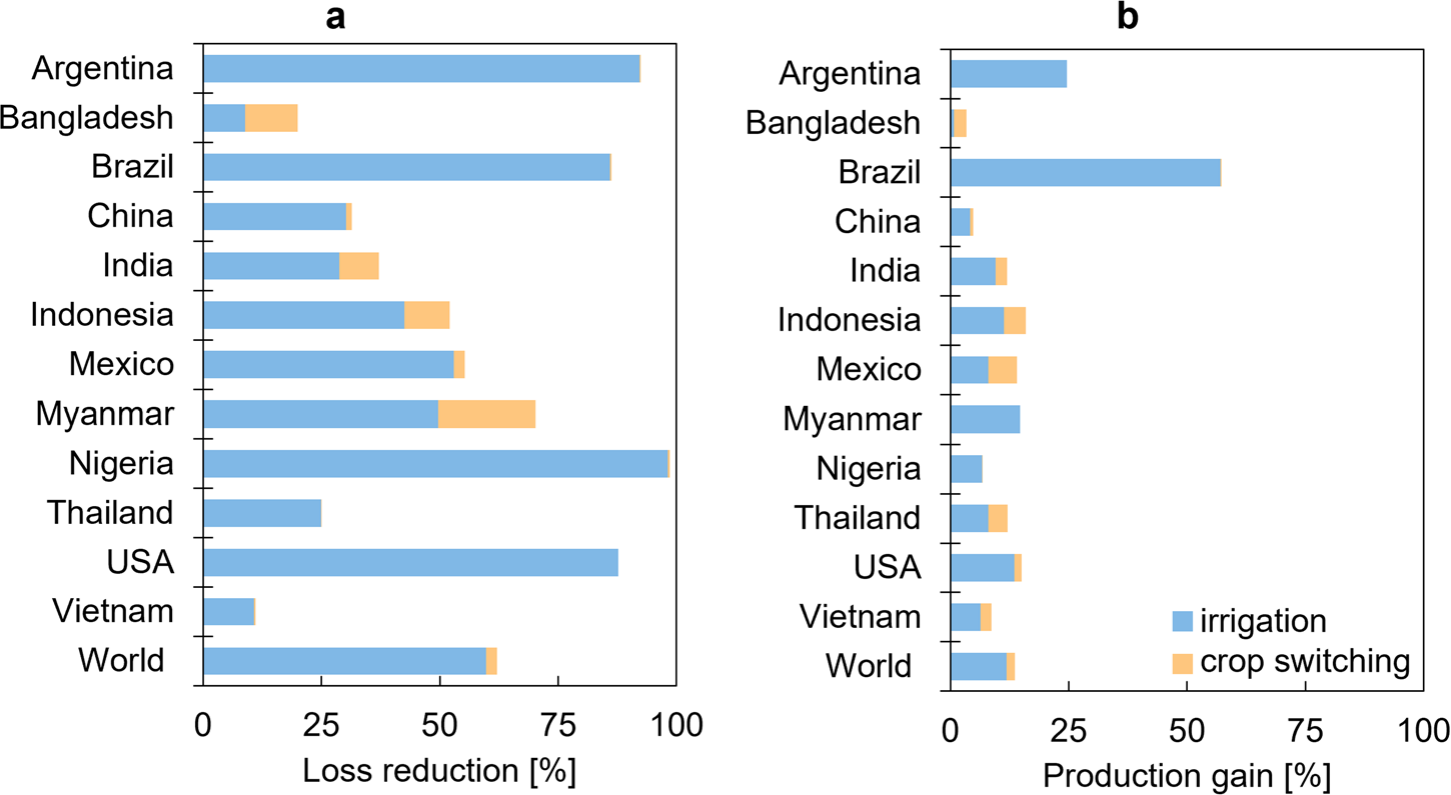
Crop production benefits of integrating sustainable irrigation expansion and crop switching. Bars show potential for reducing climate-driven production losses (**a**) and opportunities for production gains (**b**). Blue and orange bars represent the potential contribution of sustainable irrigation expansion and crop switching, respectively. The top producers of monsoon crops and the global average are shown, along with the values of expected global change. Countries are sorted in alphabetical order.

To account for the location dependence of *DS* (and therefore the varying efficacy of potential interventions), we examined spatially detailed opportunities to integrate solutions of sustainable irrigation expansion and crop switching to simultaneously buffer *DS* (by reducing production losses under extreme hydro-climatic conditions) and increase overall production (either by converting some rainfed production to irrigated or by selectively switching out crops that are both low-yielding and with high *DS*). We only allowed irrigation expansion to occur in places where there was not currently water scarcity and crop switching to occur in places where (i) an existing crop’s drought sensitivity was higher, (ii) its yield was lower than the replacing crop, and (iii) irrigation cannot be sustainably expanded due to water scarcity ^41^. Again, taking the example of monsoon cereals, we find that globally sustainable irrigation expansion alone can raise production by 12% and reduce drought-related losses by 60%, while the integration of crop switching would provide small added benefits (+2%) at the global scale. However, we observed heterogeneous effects between countries (Fig. 4). For many countries, sustainable irrigation expansion alone provided substantial benefits. The largest reductions in production losses were observed for Nigeria ( − 98%), Argentina ( − 92%), and the USA ( − 88%), where it also enables production gains through the widespread conversion to higher-yielding irrigated croplands. The largest production gains (+57%) were observed in Brazil, where drought-related production losses were also reduced by  − 86%. Substantial benefits from integrated solutions were mainly observed in countries where rice is a major monsoon crop (i.e., in South and Southeast Asia). Although sustainable irrigation expansion generally represented the majority of production changes, the crop switching further reduces production losses in Myanmar by an additional 21%, in Bangladesh, 11%, in Indonesia, 10%, and in 8%, while also achieving production gains, which are also very relevant in Mexico and Thailand. In all, this demonstrates that targeted interventions to expand irrigation and switch crops can provide large co-benefits for both climate adaptation and food supply while also ensuring water sustainability. More generally, this analysis shows the many opportunities to utilize this new drought sensitivity information to evaluate the potential crop production benefits of alternative agricultural strategies, either in isolation or in combination.

## Discussion

Climate change and variability present agricultural challenges of growing importance. Quantifying global patterns of drought sensitivity and using these to identify solutions to reduce the impacts of climate variability are critical for transitioning towards more sustainable and resilient agricultural systems. Our findings provide insights into the current drought sensitivity of global crop production and effective strategies for substantially reducing moisture-related production losses while also realizing gains in median crop production. Our analysis also develops a scale-neutral and transferable approach to quantifying the drought sensitivity of crops— measured as the percent difference in yield under median *ET*_*a*_ (i.e., typical climate conditions) and 10th percentile *ET*_*a*_ (i.e., observed extreme adverse climate conditions). This can enable the objective comparison of the drought sensitivity of different crops and contexts, the quantification of drought-related production losses, and the assessment of where potential improvements may be larger (and where actions and investments should be targeted) under a variety of agricultural interventions. Further, our drought sensitivity metric can be used to understand—from a historical perspective—whether and to what extent cropping patterns have shifted in response to climate variability (e.g., ref. ^49^) as well as whether observed cropping shifts have tended to increase or decrease the overall drought sensitivity of a particular crop or a particular country’s production basket. Similarly, our method can be applied to future scenarios of potential crop shifts to evaluate whether planned crop choice interventions—potentially focused on achieving other agricultural or sustainability objectives—may compromise the resilience and stability of crop production. Our approach can also support crop-specific high-resolution investigations of where shifting climate conditions may tend towards yield benefits (e.g., for crops currently planted in sub-optimal locations) or impacts. With all of this, our approach can effectively address key information needs of agricultural decision-makers and identify opportunities to align climate adaptation with multiple other sustainability objectives ^50^.

Our spatially explicit crop-specific analysis allowed us to identify precise hotspot locations where the drought sensitivity of crops is most pronounced and where actions may be most urgently needed to enhance the climate resilience of crop production. We find that major hotspot regions for rainfed crops exist in the US (soybean, barley, sorghum), India (wheat, soybean, cassava), central Europe (potatoes), and Brazil (potatoes, sugarcane, cassava), among other locations and crops. While a large literature has assessed the impacts of climate on staple crops (e.g., refs. ^14,51,52^) in agreement with the magnitude and spatial patterns of our estimates, our approach expands this knowledge to a much larger basket of crops (disaggregated between rainfed and irrigated) and at finer spatial resolutions. These advances provide some of the first spatially-detailed estimates of global hotspots for drought sensitivity for a suite of under-studied crops (i.e., those beyond the ‘big 4’—maize, rice, soybean, and wheat) and thereby improve the utility of such assessments in supporting climate adaptation interventions. As such, our estimates can also point to locations where economic incentives and agricultural policies may currently be promoting crops that are not well-suited agroclimatically—implying that other societal goals are being prioritized at the cost of high crop drought sensitivity.

Encouragingly, though, we find that avoided drought-related production losses under either sustainable irrigation expansion or crop switching would occur disproportionately within hotspot regions, demonstrating great promise for targeted action. With either of these solutions, it is important to note that a suite of context-specific economic, social, and environmental factors, outcomes, and feedbacks is essential to take into account before actually seeking to implement such interventions in a particular location. For irrigation expansion, it will be critical to estimate expected consumptive demand in relation to renewable availability as well as potential knock-on effects including increased competition for water use and land^53^, accompanying needs for fertilizer and other inputs^54^, impacts on water quality and environmental flows (e.g., refs. ^45,55^), alterations to local climate (e.g., ref. ^56^), displacement of smallholders^53^, and changes to food access and nutrition^57^. It is also clear that substantial investment would be required in many places in order to enable adequate access and use where water resources are relatively abundant but infrastructure is insufficient (e.g., ref. ^41^). In addition, for locations where sustainable irrigation expansion is currently possible to address drought sensitivity hotspots, it will be critical to assess the extent to which climate change may alter the patterns, volume, and timing of water availability in the future. Indeed, the extent to which irrigation can remain a solution for stabilizing and boosting crop production in certain locations depends on several factors, including increases in irrigation demand under changing climatic conditions, as well as reductions in availability (particularly surface water) through a combination of increased demand and potential alterations in rainfall patterns^44^.

In order to account for these uncertainties, sustainable expansion of irrigation to buffer crop drought sensitivity should be prioritized in locations where added irrigation demand will be well below renewable freshwater availability. For crop switching, potential effects could include changes in local food availability^58^ and farmer incomes^59,60^, altered food prices, and knock-on effects for feedstocks into agri-food supply chains (e.g., livestock, biofuels, aquaculture, etc.). Achieving widespread changes in cropping choices would also entail investments to incentivize climate-resilient crop production and to develop necessary value chains and demand^61^. While we only assess two possible strategies, our findings provide further evidence that a suite of complementary agricultural interventions can provide the largest benefits for production stability (e.g., ref. ^62^) while also achieving increased food supply. Such climate-smart and water-smart interventions include ex-situ and in-situ water harvesting, soil moisture conservation, irrigation efficiency improvements, and sustainable intensification^63,64^, all of which offer promise for increasing crop production while mitigating crop water stress. Other possible strategies include mulching techniques, managed aquifer recharge, and nature-based solutions such as contour stone bund, pitting, and terracing to increase soil moisture through enhanced infiltration rates and reduced surface runoff ^41^. Our quantification of the drought sensitivity of different crops fills a critical knowledge gap, and when combined with measurements of other dimensions of sustainability (e.g., environmental footprints, nutritional quality, profitability), can enable a spatially-explicit, quantitative, science-based evaluation of the co-benefits or tradeoffs of potential agricultural solutions (e.g., ref. ^10^).

Enhancing the climate resilience of food production can also offer benefits that propagate through food supply chains to ultimately enhance food security and nutrition^65^. Indeed, the solutions that we investigate here can simultaneously reduce climate-related production losses, increase crop production, and enhance dietary nutrient supplies—as many of the low drought-sensitivity crops also possess high nutrient contents (e.g., ref. ^66^). However, the location and magnitude of climate sensitivity for each crop can have different implications depending on the destination and purpose of the crop’s end use^67^. High climate sensitivity in areas where subsistence agriculture is practiced may mean that local food security is more vulnerable to climate risks (e.g., refs. ^68,69^). Food trade can also influence this exposure. On one hand, the food supply of import-reliant countries may be made more vulnerable through a heavy dependence on the production of a country where climate sensitivity is high (e.g., ref. ^70^). Conversely, high climate sensitivity in places that rely heavily on food imports may be buffered by increased trade volumes, thereby avoiding any substantial changes in the dietary supply of the affected crops^71^. While further investigation is needed to understand the degree to which production losses are passed through the supply chain and the extent to which climate-related production losses co-occur in time^72^, our findings provide valuable insights for exploring spatially explicit linkages between the climate vulnerability of production and the risks to places of consumption.

Lastly, decisions deriving from this study’s findings should bear in mind certain considerations regarding their appropriate use. For one, our analysis focuses on a fixed cropping extent. While this is standard practice in global crop modeling (see e.g., ref. ^51^) in order to allow like-for-like comparison between crops and regions and to isolate the effect of climate on crop yields, in reality, cropping patterns are constantly shifting^49,73^. While we do not evaluate it here, for places where temporal information on spatially detailed cropping patterns is available, our approach could be used to assess, for instance, whether cropping patterns have tended to shift away from locations where high crop drought sensitivity is measured. It is also important to note that all spatially explicit assessments are dependent on the accuracy and degree of spatial detail in the underlying agricultural statistics^74,75^. While we have compared our estimates to the best-available crop yield time series globally (see Fig. S1), recommendations deriving from ours and other spatial global products should be fully aware of these limitations when choosing appropriate scales of spatial (dis)aggregation for intervention targeting. Lastly, while our approach leverages a flexible crop water model with few parameters (e.g., management, cropping system, etc.) relative to more complex crop models (e.g., APSIM, DNDC, STICS, and DayCent), this somewhat limits the ability to explore the effect of other non-climatic factors in affecting drought sensitivity. At the same time, our approach is readily amenable to incorporating these advanced models to better understand the specific role of management practices, inputs, crop variety, and cropping systems—and various other factors determining crop productivity—on influencing the drought sensitivity of different crops. With these considerations in mind, the drought sensitivity metric developed here can serve as an important step in better quantifying and tracking the ever-changing resilience of global food production.

## Methods

Drought sensitivity (*DS*) quantifies the extent to which interannual variability in reference evapotranspiration (*ET*_0_) and precipitation affects crop yield through soil moisture anomalies and reductions in actual evapotranspiration. Reference evapotranspiration (*ET*_0_) represents the amount of water evaporated and transpired from a standardized reference crop surface. Its annual estimation integrates the surface energy balance with atmospheric evaporative demand, using meteorological variables such as net radiation, soil heat flux, air temperature, wind speed, and vapor pressure deficit. Consequently, the *DS* indicator does not directly account for the effects of temperature extremes or other non-hydrological climate stresses on crop yield; rather, it captures their influence only insofar as they alter water availability and evapotranspiration, which in turn affect yield.

*DS* is measured as the percent difference in yield under median *ET*_*a*_ (i.e., typical climate conditions) and 10th percentile *ET*_*a*_ (i.e., observed extreme adverse climate conditions) evaluated along the study period between 1961 and 2018. We do not seek to model actual yield in a given year, as this is subject to a combination of influences from climate, technology, and management practices, among other factors. Following our quantification of crop-specific *DS*, we then tested two potential solutions to reduce it: sustainable irrigation expansion and crop-switching (applied where irrigation expansion is not sustainable). We quantified for both strategies the potential reductions in climate-associated production losses as well as the potential for production gains from either increased irrigation or switches to more productive and less climate-sensitive crops. We also performed a comprehensive validation of our yield estimates in comparison with detrended official national-level yield time series and global gridded crop model outputs with constant technology and management.

### Data

A complete overview of all the data used in the study is shown in Table S1. Monthly gridded (30 arcminute or 50 km at the equator) precipitation and potential evapotranspiration data for the year 1961 through 2018 came from the CRU TS database version 4.03^76^; these data were resampled to a 5 arcminute spatial resolution to match the grid size of the other input datasets. Global gridded available water content came from the Harmonized World Soil Database (0.5 arcminute^77^)—a soil information system which integrates regional and national datasets into a consistent framework to provide standardized soil properties and classifications for use in environmental, agricultural, and climate analyses. Crop-specific and spatially explicit planting dates and length of growing period came from Portmann et al.^78^. Crop-specific irrigated and rainfed rooting depths came from ref. ^79^. Global gridded (5 arcminute or 10 km at the equator) crop-specific rainfed and irrigated harvested areas and crop yield for the year 2010 came from ^80^. We obtained a crop coefficient value for each of the four growth stages proposed by Allen et al.^79^, and we compared those values with the most up-to-date data provided by Pereira et al.^36^ on some specific locations. The proportional length of each growing stage is provided by Chapagain et al.^81^, as a function of the climatic region where the crop is grown (10 different climatic regions are considered). The yield response factor is obtained from Doorenbos and Kassam 1979, ^34^ and compared and updated with the data provided by Minhas et al. ^37^.

Crop-specific calorie content is provided in Table S4. The content is expressed in kcal per ton, and it is used to convert production losses into equivalent calorie losses.

### WaterCROP model: estimating annual crop-specific actual evapotranspiration

Actual evapotranspiration, *ET*_*a*_, (or the crop water requirement) quantifies the amount of water (in mm ⋅ day^-1^) that a crop consumes via evapotranspiration throughout the growing season. It is a function of climatic and phenological properties as well as agricultural practices (e.g., irrigated versus rainfed agriculture). We developed a gridded model derived from Tuninetti et al.^33^ to perform spatially-explicit estimates of daily *ET*_*a*,*j*_ (where *j* runs from the planting day to the harvesting day), incorporating important updates in the spatio-temporal resolution of the input data and the computational efficiency (in order to accommodate a global and time-varying assessment of crop-specific evapotranspiration). In the following, we report the main equation behind the model, while further details can be found in the original publication^33^. The spatial extent of our grid cell-level estimates corresponds to crop-specific irrigated and rainfed harvested area maps for the year 2010^80^. Annual evapotranspiration is evaluated over the study period 1961-2018 for each study crop, thus providing new data on crop water requirements, which allows new agri-hydrological analyses.

Specifically, following Tuninetti et al.^33^, daily reference evapotranspiration values (*ET*_0_) were determined through a linear interpolation of monthly *ET*_0_ data ^76^, with monthly averages assigned to the middle (i.e., the 15th day) of each month. Daily *ET*_*a*,*j*_ (*j* runs from 1 to the length of the growing period) was calculated following the FAO56 method^35,79^ for each year (over 1961–2018), where the *ET*_*a*,*j*_ estimate is equal to the product of: the daily water stress coefficient (*k*_*s*,*j*_)—a proxy for the daily water deficiency in the unsaturated soil layer; the daily crop coefficient (*k*_*c*,*j*_) which integrates the effects of crop height, crop-soil surface resistance, and albedo of the crop-soil surface^35^; and the daily *ET*_0_ from a hypothetical well-watered grass surface with fixed crop height, albedo and canopy resistance. The crop coefficient integrates for each crop the characteristics that distinguish a typical field crop from reference grass. It depends on the crop canopy and aerodynamic resistance, crop height, albedo, and evaporation from the soil. Crop coefficients vary along the growing season as the plant grows. Thus, the daily *ET*_*a*,*j*_ was calculated as:

1 $$E{T}_{a,j}={k}_{s,j}\cdot E{T}_{c,j}\cdot E{T}_{0,j}.$$

The estimate of *k*_*s*,*j*_ in each grid cell varies daily depending on the total available water content (TAWC), the readily available water content in the root zone (RAWC), where RAWC is the portion of TAWC that the crop can actually use^77,79^, and the crop-specific rooting depth (which we vary with each crop’s growing stage)^33,36^. For rainfed production, the computation of the water stress coefficient was performed through a daily steady-state water balance as in ref. ^33^. In this case, for every time step when water stored in the soil is not sufficient for optimal evapotranspiration (i.e., *ET*_*c*,*j*_ = *k*_*c*,*j*_ ⋅ *ET*_0,*j*_), the crop becomes stressed, and the water stress coefficient drops lower than 1. For irrigated production, we assumed that the crop receives all the water required to optimally evapotranspire every day (via irrigation), even when water is not available from precipitation. Hence, the water stress coefficient is equal to 1 throughout the growing period (*ET*_*a*,*j*_ = *ET*_*c*,*j*_). Taking the sum of the daily *ET*_*a*,*j*_ values for the entire growing season gave the annual *ET*_*a*_ estimate for a crop in a grid cell. This series of calculations was repeated for each grid cell, crop, and year to develop annual global gridded crop-specific estimates of irrigated and rainfed *ET*_*a*_.

In order to test the robustness of our model and the use of monthly input data to obtain annual estimates of crop actual evapotranspiration, we used ERA5 data available at both daily and monthly temporal resolutions for rainfall and reference evapotranspiration. The model was run with each dataset to estimate actual evapotranspiration for wheat over the period 1961-2018. The results demonstrate a strong agreement between the two simulations (Fig. S11), indicating little sensitivity in our estimates. Under rainfed conditions, we observed slightly higher bias and increased dispersion when using daily versus monthly rainfall data. In irrigated conditions (Fig. S11b), agreement between the two inputs was stronger, with a small bias of 7.4 mm over the cropping period. This improved agreement is expected, as rainfall plays a lesser role in irrigated systems where irrigation compensates for the moisture soil anomaly, and the observed differences are primarily due to the resolution of *ET*_0_ data.

### Evaluation of the crop drought sensitivity

The crop-specific drought sensitivity is defined as

2 $$DS=\frac{{Y}_{m}-{Y}_{10th}}{{Y}_{m}}\cdot 100,$$

expressing the percent reduction in median yield (*Y*_*m*_) associated with extreme climatic conditions (due to interannual variations in rainfall and temperature) represented by the crop yield quantified using the 10th percentile of the *ET*_*a*_ distribution evaluated in each pixel (i.e., *ET*_*a*,10*th*_, *Y*_10*th*_). The *ET*_*a*,10*th*_ is evaluated with the following approach. In each grid cell, we sorted the 58 annual *ET*_*a*_ values in ascending order and identified the 10th percentile *ET*_*a*_ (as representing historically observed extreme adverse climate conditions) and the median *ET*_*a*_ (as representing historically observed normal climate conditions). We chose the 10th percentile because it can be reliably identified with a relatively short time series (i.e., 60 years), and it is one of the most commonly used thresholds for a variety of climate assessments and indices (see e.g., ref. ^82^). We then related the 10th percentile and median *ET*_*a*_ values to yield changes via the water production function ^34,37,83^. Accordingly, a reduction of evapotranspiration causes a proportional yield reduction, whose magnitude depends on the crop-specific yield response factor (*k*_*y*_), which expresses the sensitivity of crop species to water shortage conditions. The yield response factor captures the complex linkages between production and water use by a crop, where many biological, physical, and chemical processes are involved ^34,35^. This relationship has shown remarkable validity and allowed a workable procedure for quantifying the effects of water deficits on yield ^35,37,84^. For instance, soybean has an average yield response factor of 0.85, maize 1.25, and wheat 1.05. In the *ET*-*Y* relation (see equation (3)), we calculate the *ET* change as the ratio between the year 2010 *ET* and the median *ET*_*a*,*m*_. Then, knowing the *ET*_*a*_ reduction and the 2010 crop actual yield (*Y*_2010_), we quantify the crop yield associated with the median *ET*_*a*,*m*_ (*Y*_*a*,*m*_),

3 $$\left(1-\frac{{Y}_{m}}{{Y}_{2010}}\right)={k}_{y}\cdot \left(1-\frac{E{T}_{a,m}}{E{T}_{a,2010}}\right).$$

We then repeated this calculation to determine the yield associated with the 10th percentile *ET*_*a*,10*th*_ (*Y*_10*th*_).

As such, a smaller magnitude *DS* value will indicate lower sensitivity to hydro-climatic extremes. For each grid cell, we then calculated the expected loss in production (ton) under extreme climate conditions as the product of the *DS* (percent yield loss under an extreme climate), the 2010 yield (ton per ha), and the 2010 harvested area (ha).

### Validation of the ET-Y relation to predict drought-related yield fluctuations

We adopted the ET-Y relation (equation (3)) to obtain pixel-level annual estimates of crop yield as a function of rainfall and temperature fluctuations with respect to median climate conditions. Annual yield estimates thus account for variations in the annual *ET*_*a*_ caused by fluctuations in the soil moisture availability (expressed through the *k*_*s*_ coefficient, see above) and variations in the potential evapotranspiration, and technology and management practices are held constant (circa year 2010). We validated our estimates by comparing them with the actual yield data provided at the country-level by the FAOSTAT dataset^2^ and with the ensemble-averaged outputs of LPJmL model runs (accessed through the ISIMIP platform simulation round 2a with climate models GFDL, MIROc5, IPSL, HADGEM) in which technology and management were held constant—thereby isolating the influence of climate variability on yield variations—as in our study. To compare our estimates with FAOSTAT statistics and LPJmL model outputs, we performed a pixel-wise area-weighted average of rainfed and irrigated yields. We then aggregated crop yield estimates from our study and LPJmL to national and global scales (by area-weighted averaging) to compare with FAOSTAT data. The three sets of yield time series (ours, LPJmL, FAOSTAT) were then detrended (following ref. ^85^) to obtain interannual yield anomalies, of which a large portion is known to be associated with climate variability^11^ and extremes^14^. To assess the consistency of yield responses across the three modeling frameworks, we first removed long-term linear trends from each annual yield time series using an ordinary-least-squares de-trending procedure. The resulting anomalies capture interannual variability independent of technological or management improvements. For each crop, we compared the distributional properties of detrended anomalies across models. Pairwise differences among models were evaluated using the two-sample Kolmogorov-Smirnov (KS) test, which examines whether two empirical cumulative distributions differ significantly. For each comparison, we report the KS statistic together with the test decision variable H, where *H* = 0 (false) indicates that the two model anomaly distributions are statistically indistinguishable, whereas *H* = 1 (true) indicates that the two models produce significantly different anomaly distributions (see Table S5 and Fig. S10). We note that the test was applied to all pairwise model combinations and repeated across the crops examined. When two different crops yielded identical *KS* statistics and *p*-values, this does not indicate that the crops themselves share similar anomaly behavior. Instead, it reflects that the magnitude and location of the maximum difference between the modeled and reference empirical distributions are equivalent under the KS metric. In other words, the degree of divergence between the two data sources is statistically the same for those crops, even though their underlying yield anomaly patterns may differ substantially. Overall, we find strong agreement across the study crops between the anomaly estimates derived from the ET-Y relationship and official FAO yield statistics^2^: for 12 out of 17 crops, the two sets of anomalies are statistically indistinguishable. A similarly high level of consistency is found when comparing our anomaly estimates with LPJmL outputs for most crops. We notice that LPJmL includes carbon pools and photosynthetic assimilation processes (in addition to the soil water model), and therefore represents a solid benchmark for comparison^86^. Some of the differences that we observe are likely due to the nature of the empirical ET-Y relationships that we leverage, while others may be attributable to relatively poor data quality in official statistics, particularly for lesser-studied crops^82^. We notice that for potatoes, the KS test comparisons do not indicate good agreement between our estimates and either of the two benchmarks considered. Though previous agronomic studies confirm our finding of high susceptibility of potatoes to drought stress primarily caused to their short rooting systems, which prevent them from accessing enough water during water-scarce periods^87,88^.

### Reducing drought sensitivity through sustainable irrigation expansion

Using global gridded (5-arcminute) maps of physical water scarcity ^41^, we then identified locations where rainfed crop production could be converted to irrigated crop production sustainably (i.e., without increased irrigation water demand exceeding renewable water availability). For a given grid cell where physical water scarcity was not reported, we converted all harvested areas dedicated to rainfed crops into irrigated croplands. If an irrigated yield and *DS* value for a given crop were reported for the same grid cell, we assigned those values to the converted harvested area originally allocated to the corresponding rainfed crop. For grid cells occurring in water scarcity areas for which irrigated yield and *DS* were not originally calculated but where a rainfed crop was cultivated, irrigated yield and irrigated *DS* were interpolated using an inverse distance-weighted average of surrounding irrigated grid cells. Using the newly assigned irrigated yield and irrigated *DS* value for each grid cell in areas with physical water scarcity, we were then able to quantify the reduction in production losses due to climate extremes and the increase in crop production that could potentially occur under a sustainable expansion of irrigation infrastructure (i.e., a selective conversion of rainfed fields to irrigated fields). We repeated this process using information on economic water scarcity—the condition in which freshwater resources are physically sufficient to meet irrigation water demands in a location, but where irrigation infrastructure (e.g., pumps, canals) is not currently present to enable the use of these freshwater resources.

### Crop switching to reduce production losses

We performed paired comparisons between crops to assess their relative superiority or inferiority in terms of drought sensitivity. For example, to compare the climate sensitivity of rice and maize, we first selected only grid cells where both rice and maize were cultivated to control for agro-climatic factors that may also influence yield variation. We then developed cumulative distribution function curves (CDF) for each crop, showing climate sensitivity (*x*-axis; unit: percent yield loss under an extreme climate, increasing from  − 100 to 0) versus cumulative production (*y*-axis; unit: percent of total global production for that crop). A crop with a CDF that rose less quickly moving from left to right meant that less of its production was highly sensitive to extreme climate and that, on the whole, it would experience smaller climate-related losses in production relative to the other crop. This comparison was repeated for each combination of crop pairs in the same food group in order to determine whether certain crops consistently exhibit lower relative drought sensitivity. We also performed grid-cell-level cropping changes for summer (or monsoon) cereals (i.e., rice, maize, sorghum, millet) to quantify the potential effect of crop switching on reductions in production losses from climate extremes and increases in crop production. Specifically, the harvested area of the most climate-sensitive crop in a grid cell was proportionally replaced by the other crops grown in that grid cell (based on their harvested area) whose yields were equal to or greater than the yield of the most climate-sensitive crop. This condition was to ensure that crop switches to reduce climate-related production losses would not lead to decreases in crop production. These grid-cell-level switches also helped ensure that the replacement crops could already be feasibly cultivated in that location. In doing so, we acknowledge that our analysis cannot capture field-level differences in agro-climatic characteristics, which may limit certain switches.

### Reporting summary

Further information on research design is available in the Nature Portfolio Reporting Summary linked to this article.

## Supplementary information

Supplementary Information

Peer Review file

Description of Additional Supplementary Files

Supplementary Data 1-2

Reporting Summary

## Data Availability

The results and output data generated during the current study are available from the Zenodo repository (10.5281/zenodo.18937255). All the input data used to estimate the drought sensitivity are provided in Table S1.

## Code availability

The codes developed for the analyses and to generate results are available from the Zenodo repository (10.5281/zenodo.18937255).
