# Supplementary material for: Addressing global hotspots of drought-related crop production losses
Source: Nat Commun. 2026 May 19;17:6605. doi: 10.1038/s41467-026-72715-y (PMC13381596; doi:10.1038/s41467-026-72715-y)
Supplement: Supplementary file 1 — Supplementary Information [file 41467_2026_72715_MOESM1_ESM.pdf]

# Supporting Information Materials to the paper Addressing global hotspots of drought-related crop production losses

Marta Tuninetti[a,*], Kyle Frankel Davis[b,c,d]

[a] *Department of Environment, Land, and Infrastructure Engineering, Politecnico di Torino, Turin, Italy.*
[b] *Department of Geography and Spatial Sciences, University of Delaware, Delaware, USA.*
[c] *Department of Plant and Soil Sciences, University of Delaware, Delaware, USA.*
[d] *Data Science Institute, University of Delaware, Delaware, USA.*

Table S1: Sources and spatial and temporal resolution of key data used to quantify crop-specific climate sensitivity.

| **Variable** | **Spatial resolution** | **Time coverage (resolution)** | **Source** |
|---|---|---|---|
| **Soil and climate** | | | |
| Potential evapotranspiration | 10'x10' | 1961-2018 (monthly) | CRU TS v4.03 [1] |
| Rainfall | 10'x10' | 1961-2018 (monthly) | CRU TS v4.03 [1] |
| Soil water content | 0.5'x0.5' | - | FAO [2] |
| **Agriculture** | | | |
| Planting date | 5'x5' | 2000 (monthly) | MIRCA2000 [3] |
| Length of the growing period | 5'x5' | 2000 | MIRCA2000 [3] |
| Irrigated and rainfed areas | 5'x5' | 2000 (monthly) | MIRCA2000 [3] |
| Rainfed crop yield | 5'x5' | 2010 (annual) | MAPSPAM [4] |
| Irrigated crop yield | 5'x5' | 2010 (annual) | MAPSPAM [4] |
| Rainfed harvested areas | 5'x5' | 2010 (annual) | MAPSPAM [4] |
| Irrigated harvested areas | 5'x5' | 2010 (annual) | MAPSPAM [4] |
| Yield response factor | global | - | [5, 6] |
| Stage of the growing period | regional | - | [7] |
| Rooting depth | global | - | [8] |
| Production | national | 1961-2018 (annual) | FAOSTAT [9] |
| Crop coefficient | global | - | [8, 10, 11] |

⋆

*Corresponding author
*Email address:* marta.tuninetti@polito.it (Marta Tuninetti)

Table S2: Crop-specific caloric content of the study crops.

| crop | Calories [kcal/ton] |
|---|---|
| barley | 3520000 |
| cassava | 1600000 |
| cotton | 8840000 |
| groundnut | 5670000 |
| maize | 3650000 |
| millet | 3780000 |
| oil palm | 8440000 |
| potatoes | 700000 |
| rapeseed | 8440000 |
| rice | 3600000 |
| sorghum | 3290000 |
| soybean | 4460000 |
| sugarbeet | 3870000 |
| sugarcane | 3870000 |
| sweet potatoes | 860000 |
| wheat | 3390000 |
| yams | 1180000 |

Table S3: **Results of the two-sample Kolmogorov–Smirnov (KS) tests comparing detrended yield anomaly distributions from the waterCROP model with those from FAOSTAT and LPJmL.** For each comparison, the KS statistic, the associated $p$-value, and the test decision variable H are reported. The null hypothesis ($H_0$) states that the two samples originate from the same underlying distribution. $H = 1$ (true) indicates rejection of $H_0$, implying statistically significant differences between the two anomaly distributions, whereas $H = 0$ (false) indicates that H cannot be rejected and the distributions are statistically indistinguishable. Higher $p$-values therefore denote greater similarity between the compared anomaly distributions.

| **Crop** | **Models** | **KSstats** | **pValue** | **H** |
|---|---|---|---|---|
| **wheat** | 'waterCROP vs FAOSTAT' | 0.3095 | 0.0277 | TRUE |
| **wheat** | 'waterCROP vs LPJmL' | 0.2619 | 0.0933 | FALSE |
| **rice** | 'waterCROP vs FAOSTAT' | 0.0952 | 0.9874 | FALSE |
| **rice** | 'waterCROP vs LPJmL' | 0.1667 | 0.5642 | FALSE |
| **maize** | 'waterCROP vs FAOSTAT' | 0.119 | 0.9095 | FALSE |
| **maize** | 'waterCROP vs LPJmL' | 0.2381 | 0.1588 | FALSE |
| **soybean** | 'waterCROP vs FAOSTAT' | 0.1429 | 0.752 | FALSE |
| **soybean** | 'waterCROP vs LPJmL' | 0.2857 | 0.0521 | FALSE |
| **barley** | 'waterCROP vs FAOSTAT' | 0.1429 | 0.752 | FALSE |
| **barley** | 'waterCROP vs LPJmL' | 0.2857 | 0.0521 | FALSE |
| **potatoes** | 'waterCROP vs FAOSTAT' | 0.4286 | 5.46E-04 | TRUE |
| **potatoes** | 'waterCROP vs LPJmL' | 0.4048 | 0.0013 | TRUE |
| **sugarcane** | 'waterCROP vs FAOSTAT' | 0.0952 | 0.9874 | FALSE |
| **sugarcane** | 'waterCROP vs LPJmL' | 0.2619 | 0.0933 | FALSE |
| **sugarbeet** | 'waterCROP vs FAOSTAT' | 0.381 | 0.0031 | TRUE |
| **sugarbeet** | 'waterCROP vs LPJmL' | 0.2619 | 0.0933 | FALSE |
| **COTT** | 'waterCROP vs FAOSTAT' | 0.381 | 0.0031 | TRUE |
| **COTT** | 'waterCROP vs LPJmL' | 0.2619 | 0.0933 | FALSE |
| **GROU** | 'waterCROP vs FAOSTAT' | 0.1429 | 0.752 | FALSE |
| **GROU** | 'waterCROP vs LPJmL' | 0.2381 | 0.1588 | FALSE |
| **sorghum** | 'waterCROP vs FAOSTAT' | 0.1429 | 0.752 | FALSE |
| **sorghum** | 'waterCROP vs LPJmL' | 0.2381 | 0.1588 | FALSE |
| **millet** | 'waterCROP vs FAOSTAT' | 0.2857 | 0.0521 | FALSE |
| **millet** | 'waterCROP vs LPJmL' | 0.2381 | 0.1588 | FALSE |
| **rapeseed** | 'waterCROP vs FAOSTAT' | 0.2857 | 0.0521 | FALSE |
| **rapeseed** | 'waterCROP vs LPJmL' | 0.2381 | 0.1588 | FALSE |
| **cassava** | 'waterCROP vs FAOSTAT' | 0.119 | 0.9095 | FALSE |
| **cassava** | 'waterCROP vs LPJmL' | 0.4048 | 0.0013 | TRUE |
| **sweetpotato** | 'waterCROP vs FAOSTAT' | 0.119 | 0.9095 | FALSE |
| **sweetpotato** | 'waterCROP vs LPJmL' | 0.4048 | 0.0013 | TRUE |
| **yams** | 'waterCROP vs FAOSTAT' | 0.119 | 0.9095 | FALSE |
| **yams** | 'waterCROP vs LPJmL' | 0.4048 | 0.0013 | TRUE |

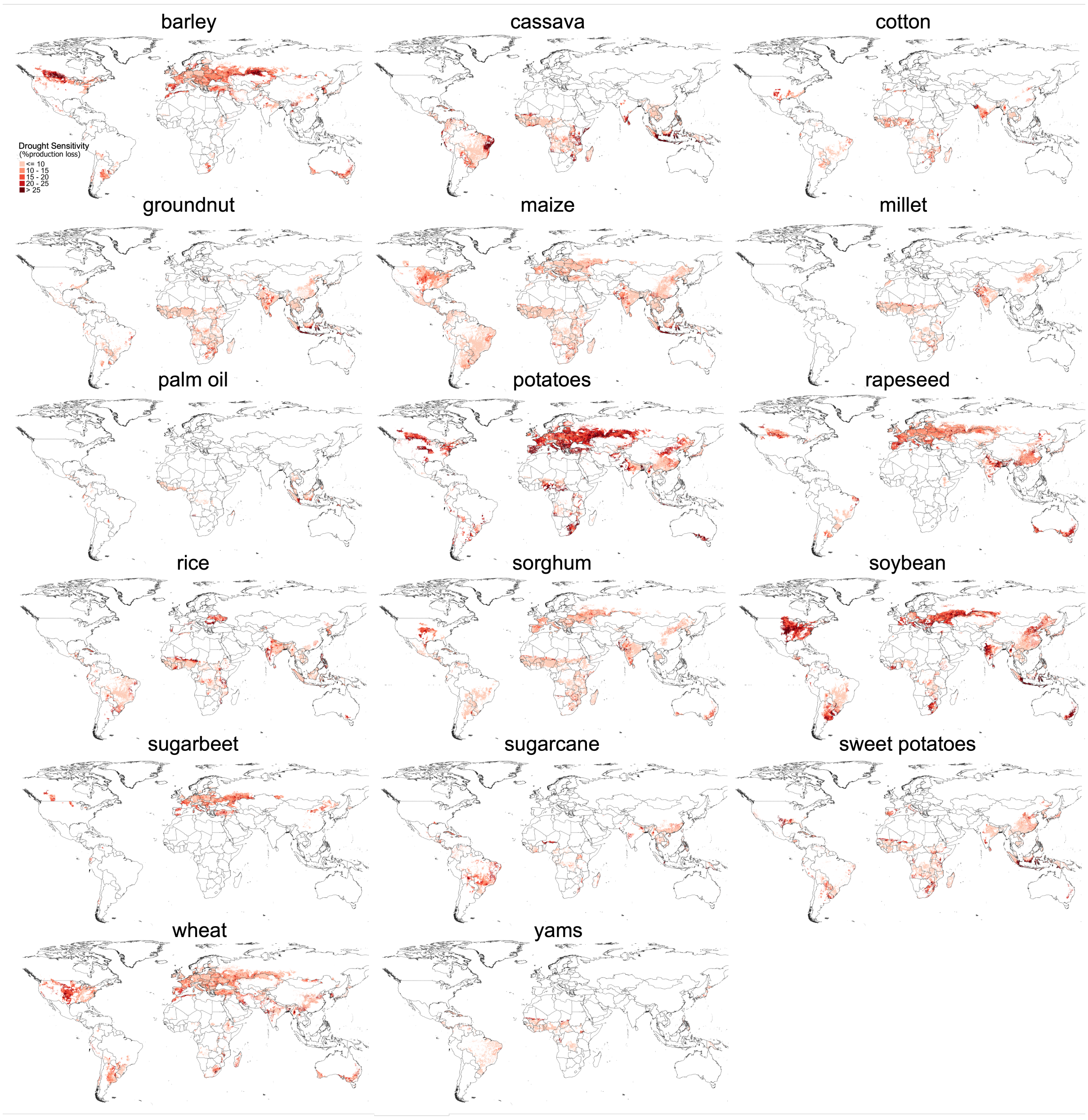


Figure S1: **Hotspots of drought sensitivity for global rainfed crop production.** Expected percent reduction in total production under extreme climate conditions are shown for each crop.

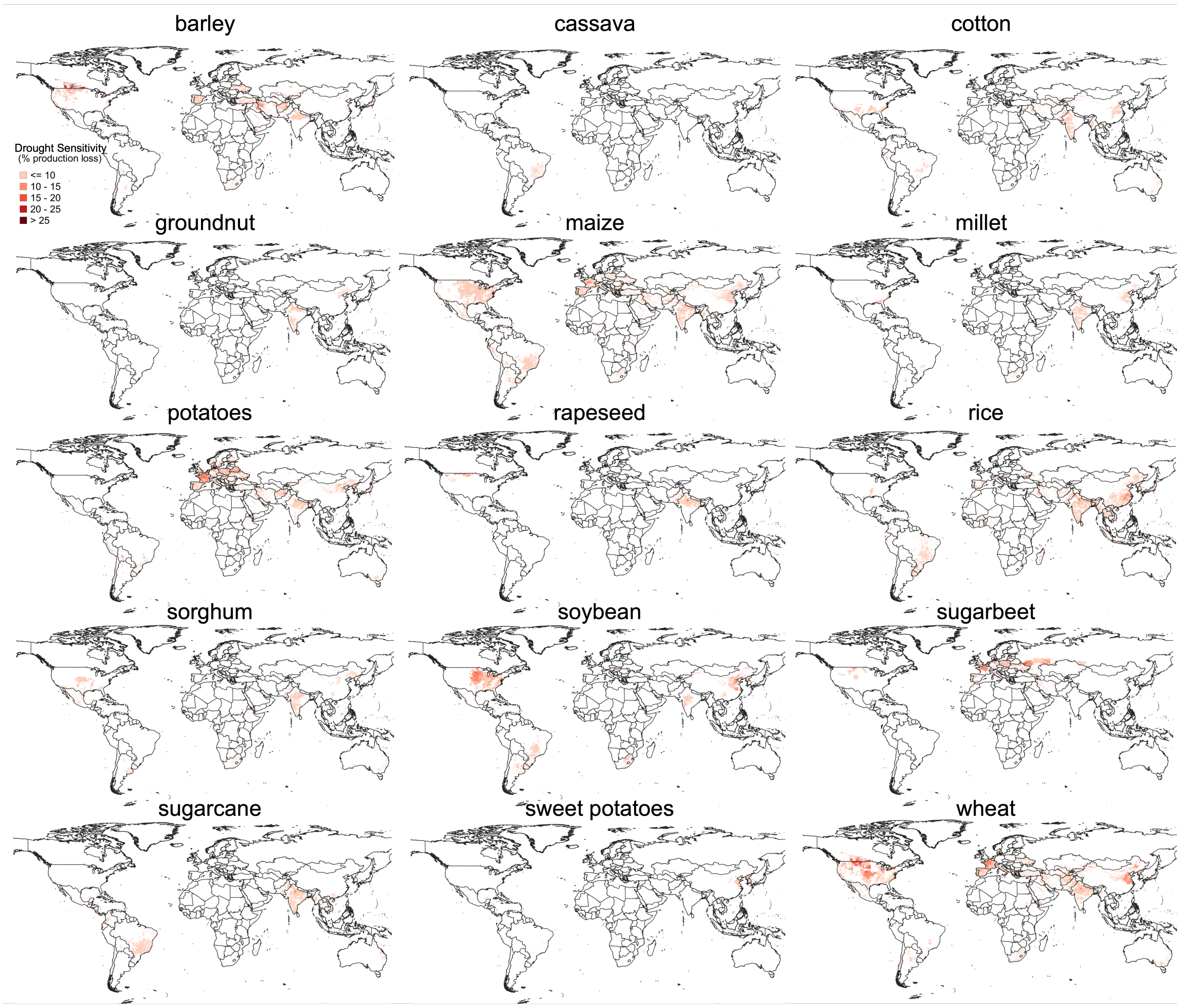


Figure S2: **Hotspots of drought sensitivity for global irrigated crop production.** Expected percent reduction in total production under extreme climate conditions are shown for each crop. Palm oil and yams are not represented as they do not show any irrigated pixels.

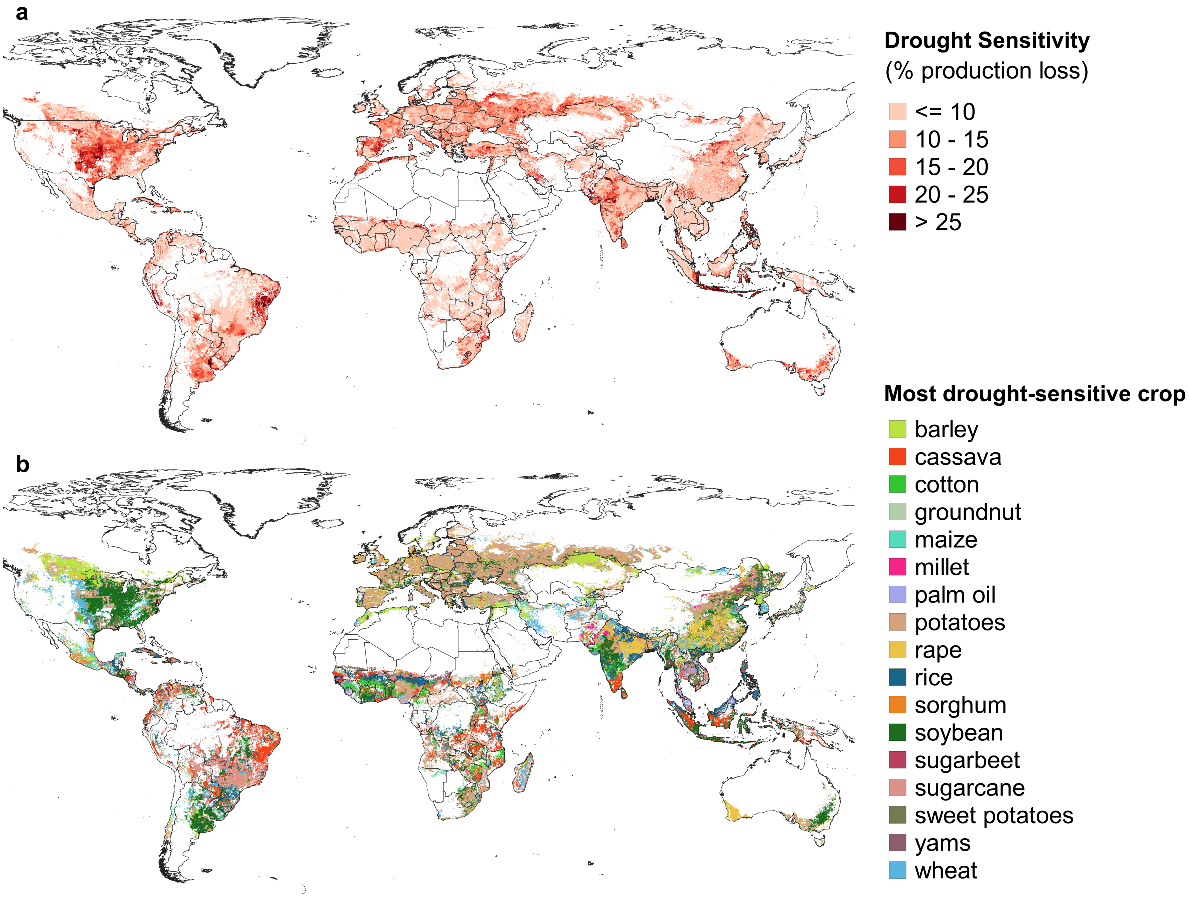


Figure S3: **Hotspots of drought sensitivity associated with the most critical cultivated crop.** Maps show locations of average drought sensitivity (a) and highlights the most sensitive crop in the pixel to extreme climate conditions (b).

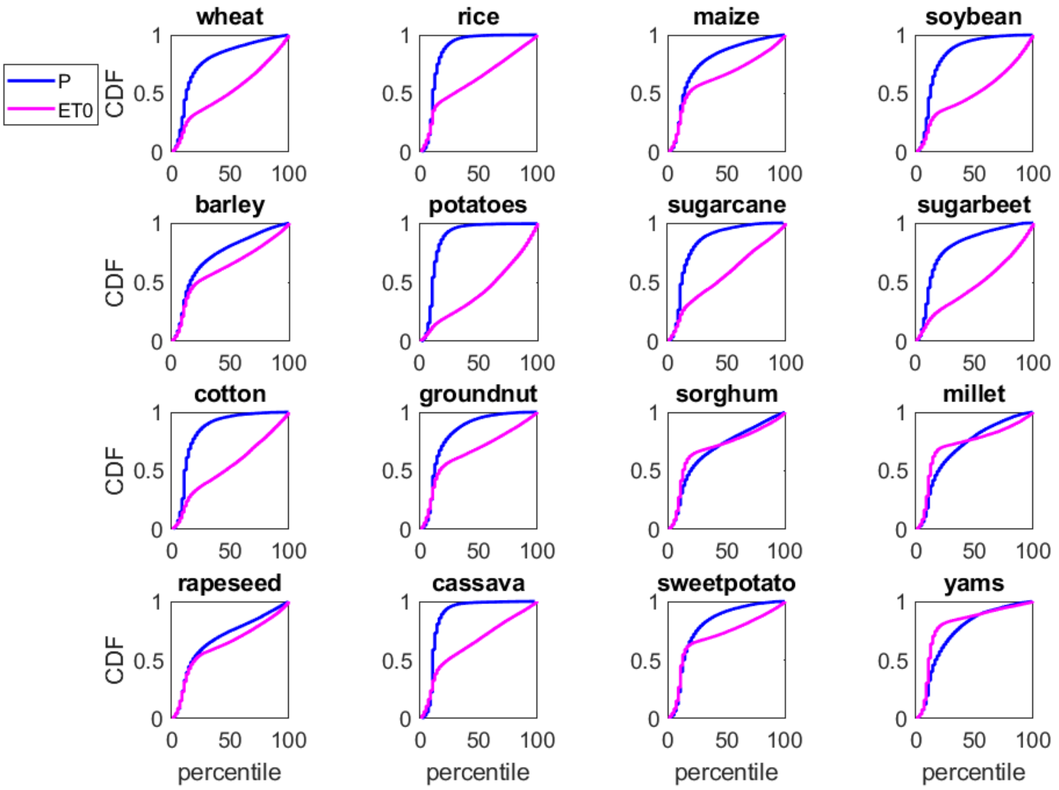


Figure S4: **Extreme precipitation and reference evapotranspiration associated to the 10th percentile $ET_a$.** Cumulative Distribution Function (CDF) of rainfall and reference evapotranspiration percentiles assessed in each pixel in correspondence of the year showing the 10th $ET_a$ percentile.

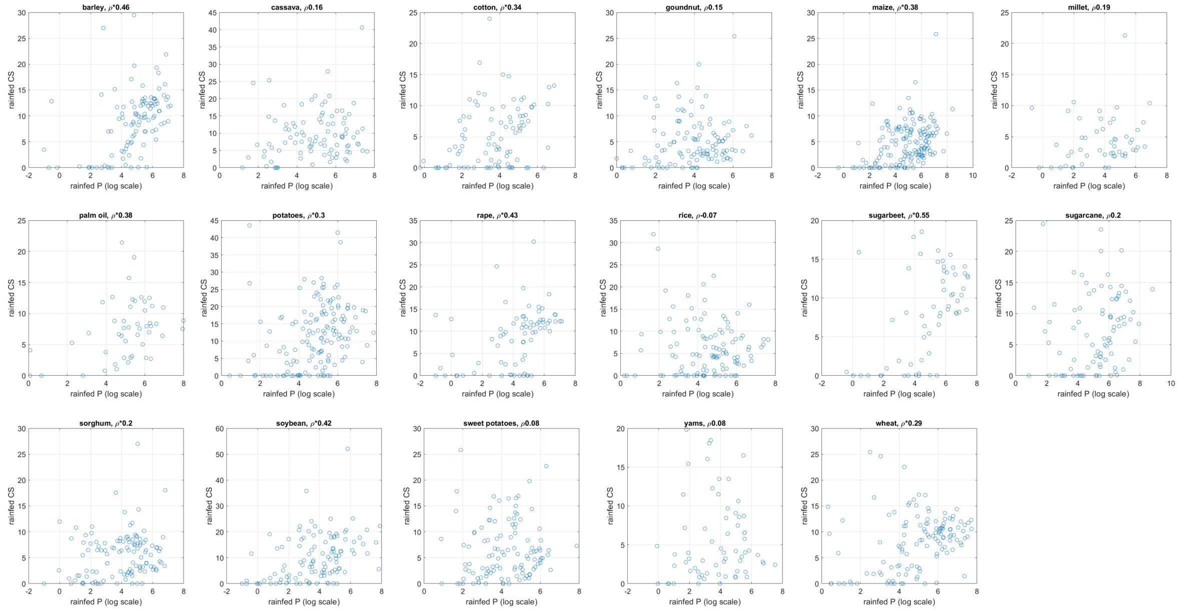


Figure S5: **Correlation between rainfed production and drought sensitivity.** Crop-specific country-average $DS$ is plotted against country rainfed production. Significant positive correlations ($p$-value 0.05) are denoted with an asterisk. Crops are shown in alphabetical order.

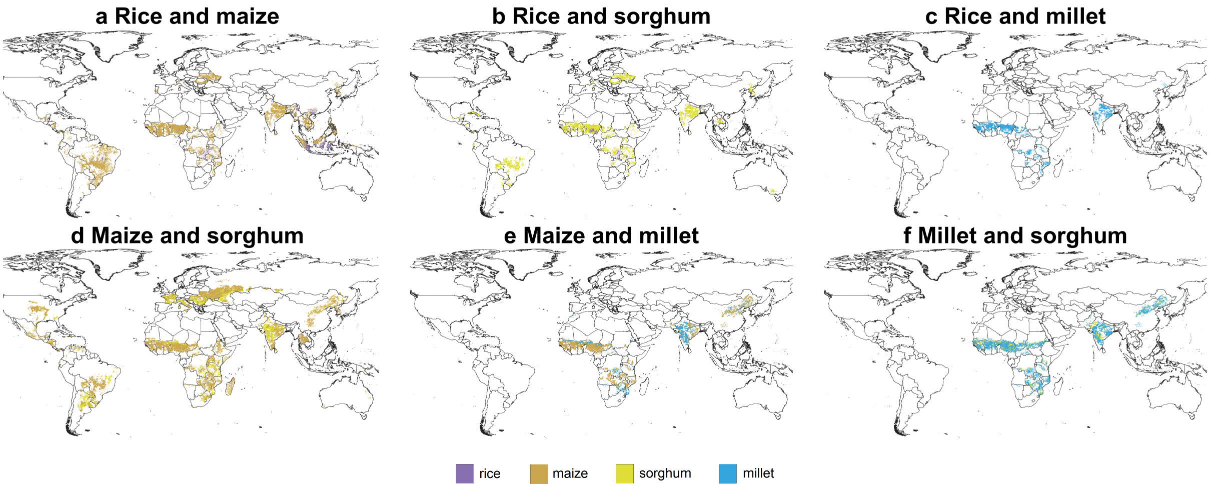


Figure S6: **Potential for drought sensitivity reduction through crop switching.** Maps show locations of co-occurence for each pair of crops: rice and maize (a), rice and sorghum (b), rice and millet (c), maize and sorghum (d), maize and millet (e), and millet and sorghum (f). Each pixel is colored with the crop having the lowest drought sensitivity and the highest yield between the pairs considered.

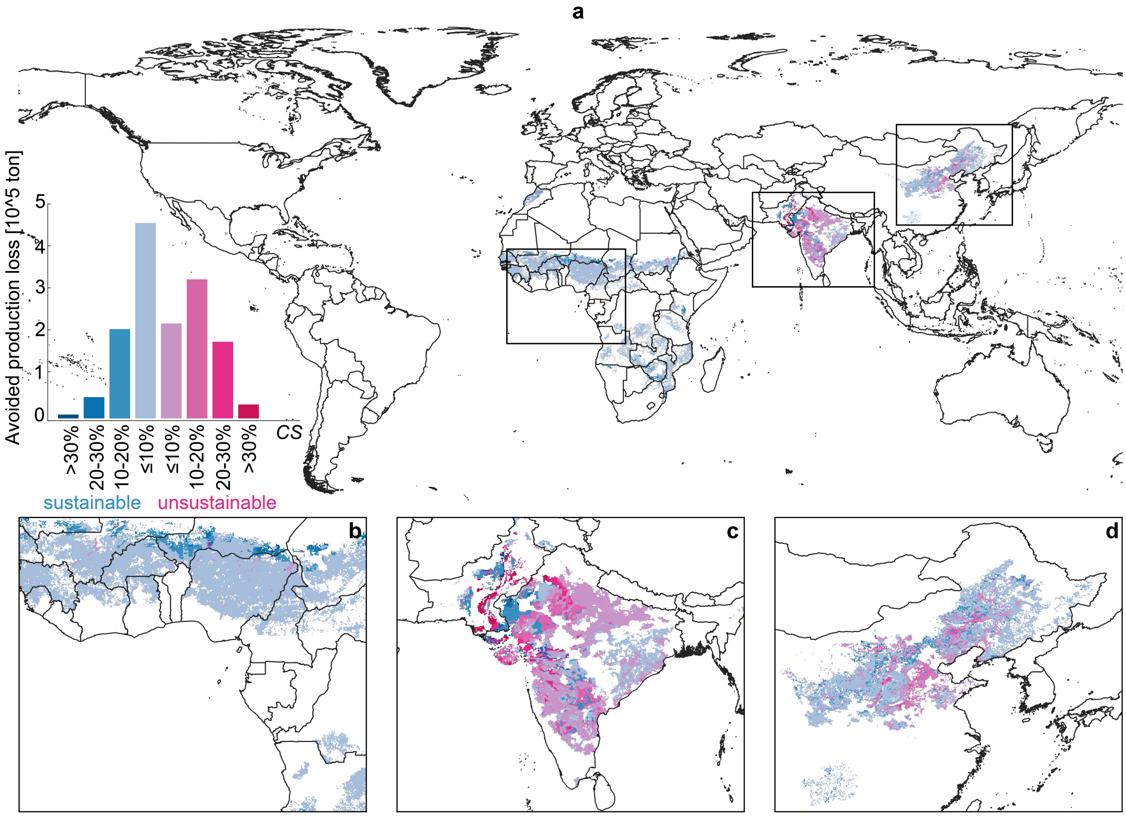


Figure S7: **Potential for drought sensitivity reduction of millet through irrigation expansion.** Map shows the drought sensitivity $DS$ of millet production and the potential for reducing it through irrigation expansion (a). Pink cells highlight the most critical hotspots of $DS$ where sustainable irrigation expansion is not possible. Blue cells indicate areas where irrigation can be sustainably expanded as a strategy to reduce $DS$. Inset histogram shows the expected avoided production loss [million ton] due to irrigation expansion. Panels are provided for several critical rice-producing areas: West Africa (b), India (c), and China (d).

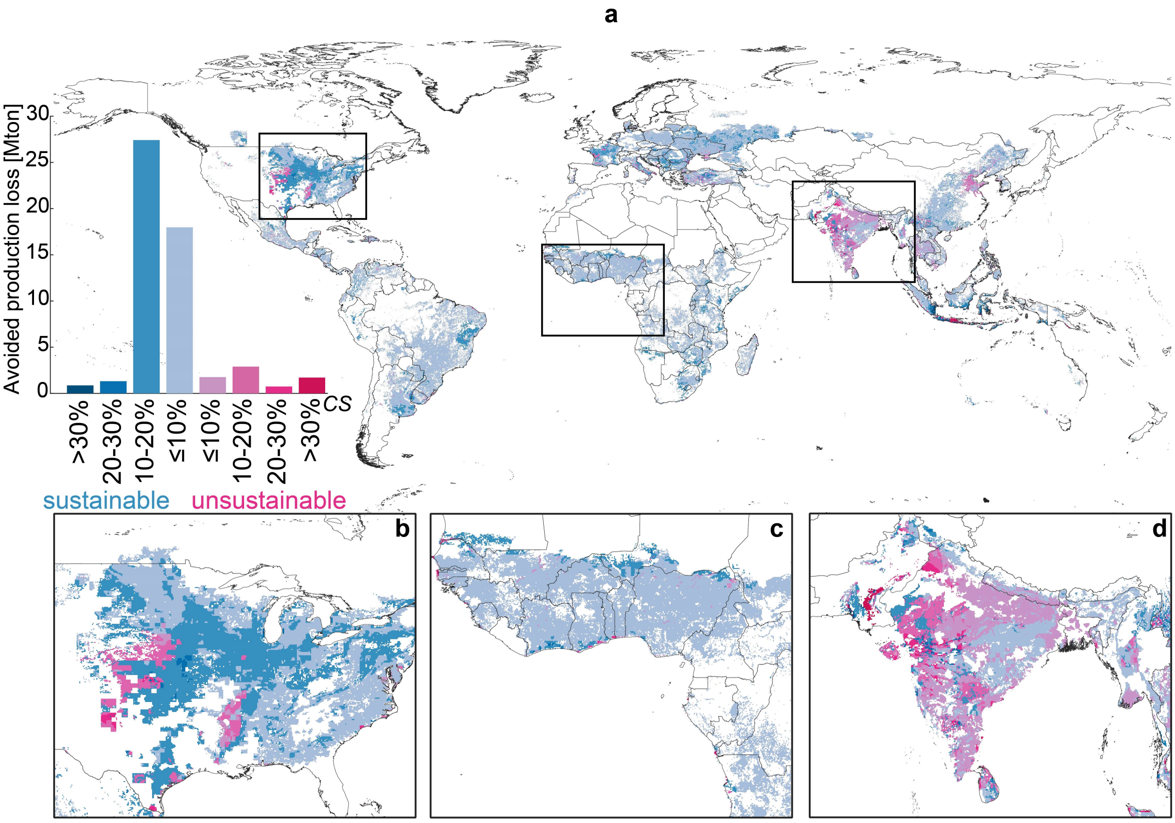


Figure S8: **Potential for drought sensitivity reduction of maize through irrigation expansion.** Map shows the drought sensitivity $DS$ of maize production and the potential for reducing it through irrigation expansion (a). Pink cells highlight the most critical hotspots of $DS$ where sustainable irrigation expansion is not possible. Blue cells indicate areas where irrigation can be sustainably expanded as a strategy to reduce $DS$. Inset histogram shows the expected avoided production loss [million ton] due to irrigation expansion. Panels are provided for several critical rice-producing areas: the USA (b), West Africa (c), and India (d).

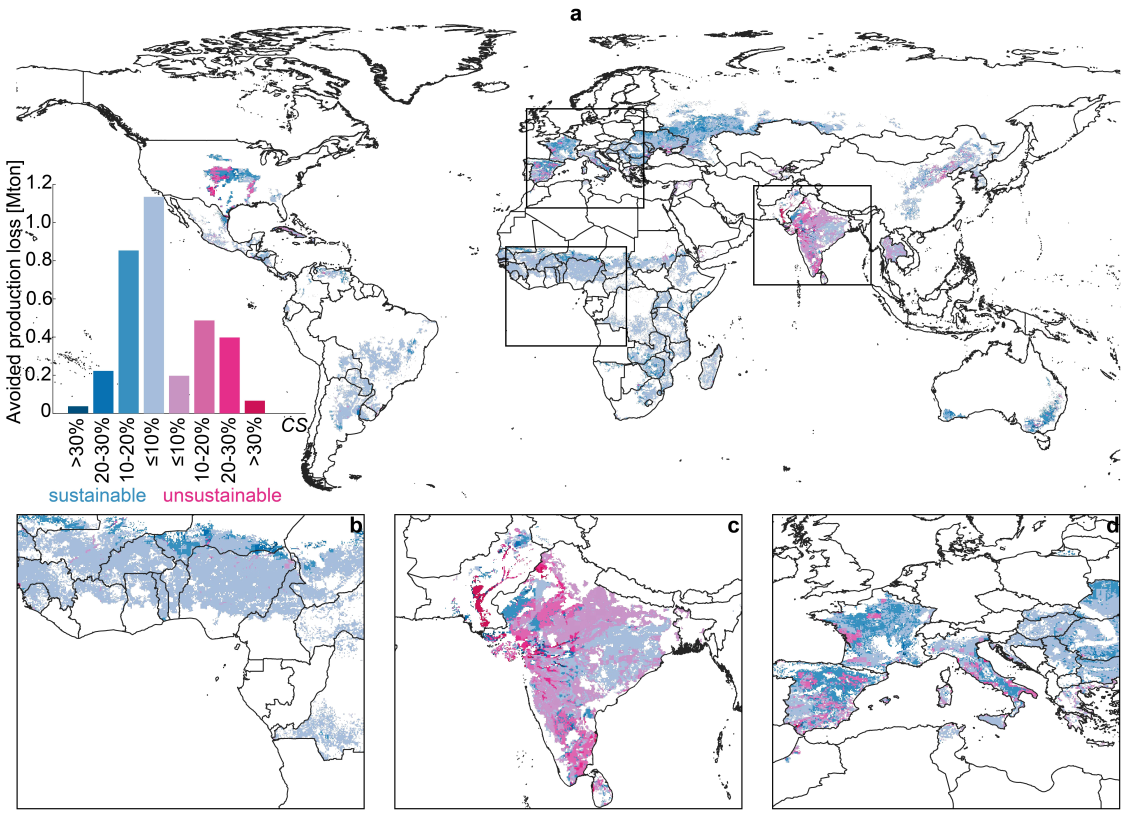


Figure S9: **Potential for drought sensitivity reduction of sorghum through irrigation expansion.** Map shows the drought sensitivity $DS$ of sorghum production and the potential for reducing it through irrigation expansion (a). Pink cells highlight the most critical hotspots of $DS$ where sustainable irrigation expansion is not possible. Blue cells indicate areas where irrigation can be sustainably expanded as a strategy to reduce $DS$. Inset histogram shows the expected avoided production loss [million ton] due to irrigation expansion. Panels are provided for several critical rice-producing areas: West Africa (b), India (c), and the Mediterranean Basin (d).

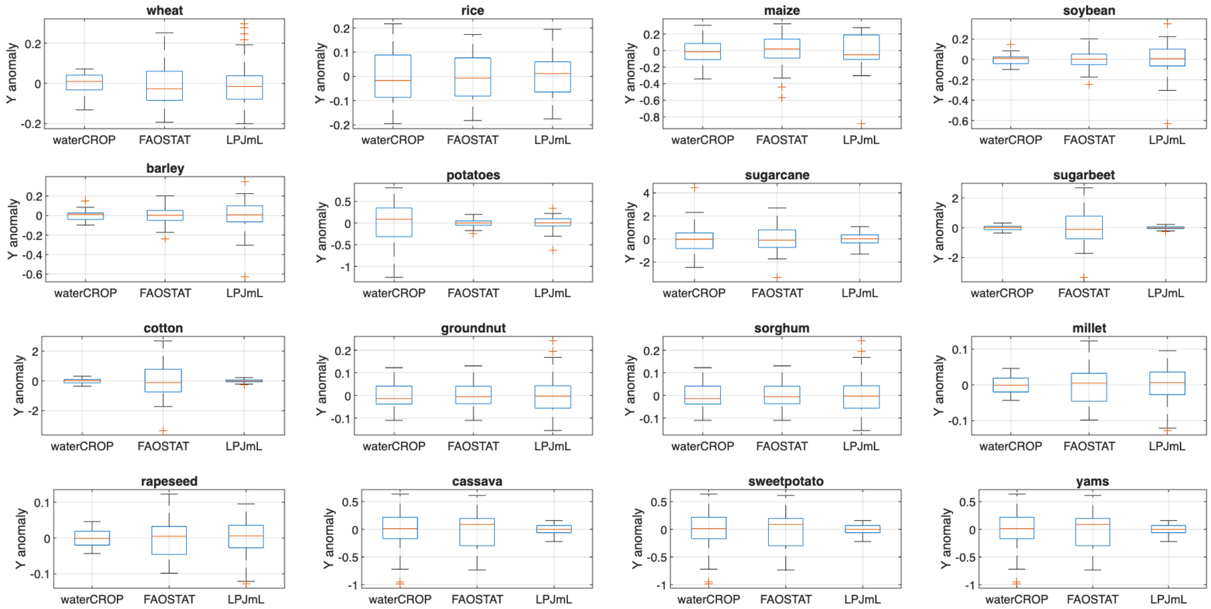


Figure S10: **Boxplots of detrended yield anomalies derived from the waterCROP model, FAOSTAT statistics, and the LPJmL model**. The anomalies represent interannual yield variability after removal of long-term linear trends. For each crop, the boxplots illustrate the median, interquartile range, and distribution tails of the anomaly series.

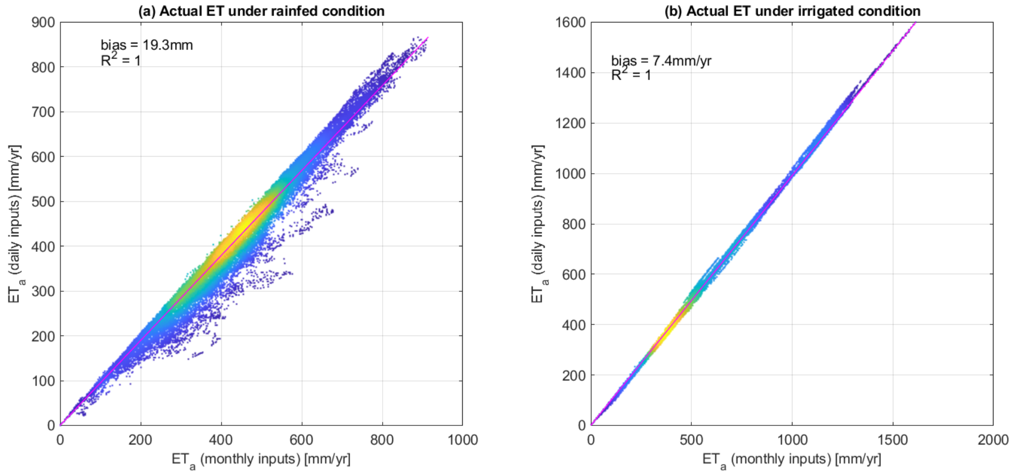


Figure S11: **Testing the temporal resolution of the input data to the WaterCROP model.** Comparison between the actual crop evapotranspiration obtained for wheat using monthly (x-axis) and daily (y-axis) input data under rainfed (a) and irrigated (b) conditions.

## References

[1] I. Harris, T. J. Osborn, P. Jones, D. Lister, Version 4 of the cru ts monthly high-resolution gridded multivariate climate dataset, Scientific data 7 (1) (2020) 1–18.

[2] FAO, IIASA, ISRIC, ISSCAS, JRC, Harmonized world soil database: http://webarchive.iiasa.ac.at/research/luc/external-world-soil-database/html (2012).

[3] F. T. Portmann, S. Siebert, P. Döll, Mirca2000—global monthly irrigated and rainfed crop areas around the year 2000: A new high-resolution data set for agricultural and hydrological modeling, Global biogeochemical cycles 24 (1) (2010) GB1011.

[4] IFPRI, Global Spatially-Disaggregated Crop Production Statistics Data for 2010 Version 2.0 (2019). `doi:10.7910/DVN/PRFF8V`.

[5] J. Doorenbos, A. Kassam, C. Bentvelsen, Yield response to water, FAO irrigation and drainage paper, Food and Agriculture Organization of the United Nations, 1979.

[6] P. Minhas, T. B. Ramos, A. Ben-Gal, L. S. Pereira, Coping with salinity in irrigated agriculture: Crop evapotranspiration and water management issues, Agricultural Water Management 227 (2020) 105832.

[7] A. K. Chapagain, A. Y. Hoekstra, Water footprints of nations (2004).

[8] R. G. Allen, L. Pereira, D. Raes, M. Smith, Fao irrigation and drainage paper no. 56, Rome: Food and Agriculture Organization of the United Nations (1998) 26–40.

[9] FAO, Faostat: http://faostat.fao.org (2014).

[10] L. Pereira, P. Paredes, N. Jovanovic, Soil water balance models for determining crop water and irrigation requirements and irrigation scheduling focusing on the fao56 method and the dual kc approach, Agricultural water management 241 (2020) 106357.

[11] L. Pereira, P. Paredes, D. Hunsaker, R. López-Urrea, Z. M. Shad, Standard single and basal crop coefficients for field crops. updates and advances to the fao56 crop water requirements method, Agricultural Water Management 243 (2021) 106466.
