# Supplementary material for: Addressing global hotspots of drought-related crop production losses
Source: Nat Commun. 2026 May 19;17:6605. doi: 10.1038/s41467-026-72715-y (PMC13381596; doi:10.1038/s41467-026-72715-y)
Supplement: Supplementary file 3 — Description of Additional Supplementary Files [file 41467_2026_72715_MOESM3_ESM.pdf]

Description of Additional Supplementary Files

File Name: Supplementary Data 1
Description: Countryaveraged drought sensitivity for crops grown under rainfed conditions.

File Name: Supplementary Data 2
Description: Countryaveraged drought sensitivity for crops grown under irrigated conditions.
