# Supplementary material for: Addressing global hotspots of drought-related crop production losses
Source: Nat Commun. 2026 May 19;17:6605. doi: 10.1038/s41467-026-72715-y (PMC13381596; doi:10.1038/s41467-026-72715-y)
Supplement: Supplementary file 2 — Peer Review file [file 41467_2026_72715_MOESM2_ESM.pdf]

nature portfolio

Peer Review File

Addressing Global Hotspots of Drought-related Crop Production Losses

Corresponding Author: Professor Marta Tuninetti

This file contains all reviewer reports in order by version, followed by all author rebuttals in order by version.

Version 0:

Reviewer comments:

Reviewer #1

(Remarks to the Author)
Using historical climate data and process-based crop models, the authors evaluate the climate sensitivity of 17 crops at the grid cell level, which they define as the difference between median yields and yields at the 10th percentile of the evapotranspiration distribution, expressed as a percentage of median yield. They identify hotspots of climate sensitivity with respect to geography and crop type and then evaluate the potential for irrigation expansion and crop switching to reduce climate-related crop losses. This study is well-written, provides new insights into spatial and crop-specific patterns of climate sensitivity, and explores important solutions for improving resilience. The methods for evaluating crop switching benefits are particularly creative and compelling. I have one major concern and a handful of minor concerns that I believe can be addressed with revisions.

Major comments:

The term “climate sensitivity” is used throughout to refer to the percentage reduction in yield that occurs relative to median at the 10th percentile of cumulative seasonal crop ET based on crop model output forced with historical climate data. Based on the calculation of ETa, it seems that this definition of “climate sensitivity” will effectively capture crop stress related moisture-limited conditions, but not necessarily stress related to direct effects of hot or cold temperatures, waterlogging, or shifts in seasonality. The monthly timestep of the climate data further limits the ability of the analysis to capture stress related to short-term moisture limitation and heat stress. Water limited conditions are only responsible for a fraction of total climate-related production losses, and thus, I think that the use of the term “climate sensitivity” and associated phrases like “production losses under extreme climatic conditions” are somewhat overstated and potentially misleading to a cursory reader. This clarification is particularly important in the text discussing the benefits of sustainable irrigation expansion (especially related to Fig 4a), which currently (overoptimistically) implies that irrigation expansion could eliminate 60% of total global climate-related crop losses and >80% of climate-related crop losses in Argentina, Brazil, Nigeria, and the USA. I suggest three revisions related to this issue:

1. The “climate sensitivity” and “extreme climate” terminology should be altered to be more specific, and text should be added to the last paragraph of the introduction and/or the discussion to clarify the types of climate stressors that this analysis can and cannot capture.
2. It would be informative to report the climate conditions under which the 10th percentile ET conditions occur to demonstrate that they are in fact extremes. For example, what are the median temperature and precipitation percentiles associated with the 10th percentile ET? Perhaps the T and P percentiles associated with the 10th percentile of ET could be mapped or the correlations between ET percentiles and climate percentiles plotted to show that low ET is indeed occurring in years with extreme climate conditions.
3. Monthly climate data are linearly interpolated to the daily timestep and used as model input. The use of monthly data precludes capturing the effects of shorter-term climate extremes and stressors. I suggest using daily climate data directly, instead of interpolated monthly data.

Minor comments:

Line 23: the phrase “spatially detailed patterns” could be deleted to reduce redundancy with the following line (“at fine spatial granularity”).

Line 31: Please provide a reference for the statement that these crops constitute 75% of production.

Line 50-51: Typos, correct “w” to “we” and delete redundant “here” after “of rainfed production”

Lines 54-55 (and associated abstract text): These lines state the caloric impact of production losses, but I do not see information in the methods about how production losses were converted to calories. Please add the conversion details to the methods.

Line 60: No SI maps are provided for irrigated palm oil or yams; is there no irrigated area for these crops?

Lines 69-71: What is the unit used for rainfed production in these correlations (hectares, tonnes, calories)? Please specify in the SI legend. Also, change “18 crops” to “17 crops”.

Line 76: In addition to not allowing irrigation expansion into areas currently experiencing water scarcity, is the irrigation expansion scenario limited to areas that will be able to support all projected additional irrigation without water scarcity?

Line 113: Acronyms for sustainable irrigation expansion and crop switching are introduced here, after the results sections for each of those solutions that did not use acronyms. I recommend omitting the acronyms entirely. If they are retained, introduce them from the first use of each term.

Lines 158-229: I suggest splitting up these very long paragraphs to improve readability. As-written, natural breaks could occur at lines 170, 185, and 215.

Line 141-150: It would be helpful to have some additional context for how the climate sensitivity results from this paper compare with the existing literature on climate impacts on crops in terms of magnitude, spatial patterns, crop-specific differences, etc.

Line 208: Missing closing parentheses

(Remarks on code availability)

Reviewer #2

(Remarks to the Author)
I co-reviewed this manuscript with one of the reviewers who provided the listed reports. This is part of the Nature Communications initiative to facilitate training in peer review and to provide appropriate recognition for Early Career Researchers who co-review manuscripts.

(Remarks on code availability)

Reviewer #3

(Remarks to the Author)
• What are the noteworthy results?

This paper presents an interesting contribution by assessing global crop climate sensitivity and identifying hotspots vulnerable to climate variability. This approach offers insights for enhancing agricultural resilience in climate-sensitive regions, aligning adaptation strategies with food security goals. A new methodology is proposed to evaluate the effect to expand “sustainable irrigation” and evaluating the possibility offered by a crop switching. The work is impressive from the point of view of the data collection and for the ability to manage a very high number of cells, crops and years.

• Will the work be of significance to the field and related fields? How does it compare to the established literature? If the work is not original, please provide relevant references

This work is relevant in its attempt to identifying at very detailed scales regions and hotspots where the sensitivity to climate of the crops is hight, and where is possible to increase yield stability using irrigation or ‘sustainable irrigation’. The proposed methodology is new and interesting. This approach expands the scale and detail available in previous literature, that are limited to major crops at country scale. Established literature is considered and the reference are up to date.

• Does the work support the conclusions and claims, or is additional evidence needed?

The work supports its conclusions and claims. However, at least in our opinion, the approach based only on an evaluation of ETa along the year, followed by a set of empirical relations linking ETa to yield losses may be well explained. In this work the soil is only a water reservoir, not a resource that could be compromised (or improved) with different cropping system and relative management. Other important limitation to the productivity as those linked to nutrient availability are not considered. At this detailed scale actually seem very difficult to use cropping system models that consider also other alternative, as

nutrient management, tillage option, use of cover crops and so on, but we think that we have to avoid giving the impression that the performance of an agricultural system depends only on the temperature and water. Also, some consideration about extreme events must be done. We think that other cropping system models that include soil dynamics of nutrient, and evolution of organic carbon need to be mentioned. (examples are Daycent, Stics, Armosa, DNDC, APSIM etc…)
• Are there any flaws in the data analysis, interpretation and conclusions? Do these prohibit publication or require revision? mmmm

The data analysis and interpretation have some limitations that would benefit from revision to improve the study's rigor but do not prohibit publication. As previously stated, the study claims a process-based approach, but yield modelling relies on empirical functions rather than true process-based yield simulations. The readers have to be conscious of this approach. We think that this potential limitation need to be highlighted, and the text where is supported the quality of this approach comparing statistical data with model result need to be improved.
May be that the recommendations for sustainable irrigation expansion and crop switching are promising but may oversimplify regional variability. For instance (see after) look at the Mediterranean rice's sensitivity, that seem not dependent from water but from critical temperatures during the crop development .
We think that the comparison with already existing model need to be enforced and not limited to the correlation coefficient. We think that some revision is needed, but the general validity of the work is not under discussion, and there not flaws that prohibit the publication of the manuscript after revision.

• Is the methodology sound? Does the work meet the expected standards in your field?

The methodology is promising and is applied in a clear and reproducible way. There are some minor issue, described in the list of critical point to be addressed.

• Is there enough detail provided in the methods for the work to be reproduced?

Yes, all the detail to reproduce the methodology and to reproduce the work are adequately described and supported, also with good supplemental material.

Specific comments
Abstract
• "combining gridded climate data, spatially-explicit agricultural statistics, and process-based crop modeling to quantify global patterns of rainfed and irrigated crop climate sensitivity" in our opinion the term process-based should be substituted
• "We estimate -10.1% and -6.8% losses in global rainfed and irrigated production (respectively) under historically observed extreme climate conditions" having used historical data, it is unclear in relation to which productivity values the losses have been calculated
• "Our new scalable and universal approach" due to the approach limitations listed in the previous comments, we belive that the term universal should be substituted
• Line 21-23: we belive it would be useful to also mention the application case study, and limitations, of global gridded crop models or the application of process-based model at a national or continental scale in this section (it might be helpful to also move your reference [62] (Müller et al., 2017) here)
Arboleda-Obando, P. F., Ducharne, A., Yin, Z., and Ciais, P.: Validation of a new global irrigation scheme in the land surface model ORCHIDEE v2.2, Geosci. Model Dev., 17, 2141–2164, https://doi.org/10.5194/gmd-17-2141-2024, 2024
Bassu, S., Brisson, N., Durand, J.-L., Boote, K., Lizaso, J., Jones, J.W., Rosenzweig, C., Ruane, A.C., Adam, M., Baron, C., Basso, B., Biernath, C., Boogaard, H., Conijn, S., Corbeels, M., Deryng, D., De Sanctis, G., Gayler, S., Grassini, P., Hatfield, J., Hoek, S., Izaurralde, C., Jongschaap, R., Kemanian, A.R., Kersebaum, K.C., Kim, S.-H., Kumar, N.S., Makowski, D., Müller, C., Nendel, C., Priesack, E., Pravia, M.V., Sau, F., Shcherbak, I., Tao, F., Teixeira, E., Timlin, D. and Waha, K. (2014), How do various maize crop models vary in their responses to climate change factors?. Glob Change Biol, 20: 2301-2320. https://doi.org/10.1111/gcb.12520
Dokoohaki, H., Kivi, M. S., Martinez-Feria, R., Miguez, F. E., & Hoogenboom, G. (2021). A comprehensive uncertainty quantification of large-scale process-based crop modeling frameworks. Environmental Research Letters, 16(8), Article 084010. https://doi.org/10.1088/1748-9326/ac0f26
Archontoulis SV, Castellano MJ, Licht MA, et al. Predicting crop yields and soil-plant nitrogen dynamics in the US Corn Belt. Crop Science. 2020; 60: 721–738. https://doi.org/10.1002/csc2.20039
Folberth C, Elliott J, Müller C, Balkovič J, Chryssanthacopoulos J, et al. (2019) Parameterization-induced uncertainties and impacts of crop management harmonization in a global gridded crop model ensemble. PLOS ONE 14(9): e0221862. https://doi.org/10.1371/journal.pone.0221862
• Line 29-30: Is it possible to indicate the source of the list of crops that make up 75% of global primary production? Wouldn't it be more useful to list these crops in descending order of productivity?

• Line 32: Is it possible to provide more detail concerning this daily process-based crop-water model?
• Line 36: can you elaborate on how the approach employed to estimate the effects of salinity on crop ET and yields (reference [26]) is modified to be integrated in your approach?
• Line 38: It is not very clear why the extreme adverse climate conditions are identified when ETa is below the 10th percentile
• Line 50: Here we focus
• Line 51: "here" is repeated again
• Line 53: check the brackets
• Line 54-55: this sentence, which provides information about irrigated crops in parentheses, is not very smooth to read

• Line 57-58: It should be emphasized that the CS values for these crops confirm information already known from an agronomic point of view
• Line 80-83: It may be argued that this example is not applicable, particularly in Mediterranean regions. In the Mediterranean Basin, nearly all rice cultivated is paddy rice, with very limited areas irrigated using alternative methods. Rainfed rice does not exist in this context. For rice cultivation, climate sensitivity is primarily attributed to lower temperatures during critical phenological stages of crop development, especially during the flowering phase, rather than the amount of water available.

• Line 115-118: The criteria for crop switching (CrSw) are not written in a very fluid and clear manner. In particular, criterion (i) could be reformulated to enhance clarity
• Line 120: reduce losses by 60%
• Line 126-127: Is it possible to elaborate on the reasons?
• Line 146: we belive that SIE and CrSw do not constitute a variety of agricultural intervention, rather, they represent 2 possible strategies that have been analyzed
• Line 170-174: Perhaps a statement regarding the implications of sustainable irrigation expansion on other components of the cropping system (such as nitrogen) should be included, as these aspects are not addressed at all here. This addition would facilitate an acknowledgment and an assessment of the limitations associated with this type of approach
• Line 231-233: This statement should be revised as it is misleading and reflects a reductionist approach. Solar radiation, along with other driving variables affecting agro-ecosystems, plays a crucial role. In particular, radiation is a key variable for estimating ETa.
• Line 238: Please define the term potential evapotranspiration (PET). The FAO 56 irrigation and drainage paper propose to avoide the use of the term PET due to its potential for varied interpretations. Instead, please use ET_0 to refer to reference evapotranspiration and ETc to refer to crop (maximum) evapotranspiration
• Line 241-243: Please, show this comparison in some supplemental material.
• Line 254: Would it be possible to include a brief description of the principles behind the database construction
• Line 260-XX: “We developed a gridded algorithm derived from Tuninetti et al. [22]” even if the algorithm is taken from another publication it could be interesting to shortly describe the alorithm here
• Line 265: Is root depth variable during phenological stages?
• Line 267: When the term 'water from precipitation' is used, it may be more accurate to refer to water stored in the soil? This phrasing suggests a direct relationship between rainfall and stress, without acknowledging the mediating role of soil water content
• Equation 2 and 4: Is it necessary to repeat the same equation for calculating CS twice?
• Line 288-289 and 295-296: reference format to be fixed
• Line 298-300: Please provide a brief explanation of why there is a need for conducting a time series correlation analysis. This sentence and the following one is unclear and needs to be reformulated.
• Line 300: Please replace “relationship” with correlation
• Line 301-303: Could you briefly explain in detail why the shift was implemented?
• Line 304: It may be worthwhile to comment on the fluctuations of the model for the crops cassava, rice, and sugarcane, as shown in Extended Data Figure 2
• Line 309: Supplementary Figures S38-S42
• Line 339: It seems that the Extended Data Figures are not cited in the text according to their numbering, so it may be necessary to rearrange them
• Figure 1: It may be advisable to reconsider the font sizes on the horizontal axes labels of the bar plots dedicated to the countries. Additionally, it is unclear how these 6 countries featured in the bar plots were selected, can you elaborate in the figure caption?

(Remarks on code availability)

Reviewer #4

(Remarks to the Author)
I co-reviewed this manuscript with one of the reviewers who provided the listed reports. This is part of the Nature Communications initiative to facilitate training in peer review and to provide appropriate recognition for Early Career Researchers who co-review manuscripts.

(Remarks on code availability)

Reviewer #5

(Remarks to the Author)
Gridded climate data, spatially-explicit agricultural, statistics, and the Watercrop model based on a simple yield-ET function, were combined to quantify global patterns of rainfed and irrigated crop climate sensitivity and climate-associated production losses for 17 major crops. A -10.1% and -6.8% losses in global rainfed and irrigated production was estimated under historically observed extreme climate conditions with hotspots of climate sensitivity in the central US, eastern Brazil, the Mediterranean basin, and South Asia. To illustrate how sustainable irrigation expansion and targeted crop switching could

reduce climate sensitivity, a case was introduced for rice, maize, millet, sorghum replacing each other and it was shown that 62% of production losses could be avoided while increasing overall production by 14%. It was concluded that this approach in measuring climate sensitivity of crops enables the assessment of where climate-related production losses tend to be largest and where mitigating actions and investments can be proactively targeted to better ensure the stability and increased supply of global crop production.

While this is an interesting idea of exploring the potential of irrigation where water is sustainable availability and crop switching to reduce the impact of season-to-season variability of low yields on global food production, the applied Watercrop model seems an unsuitable tool this task. The validation of comparing the Watercrop model with another benchmark model and FAO yield data shows mostly no correlation and in some cases high yields when the other model or FAO yield data show low yields and visa versa. The Watercrop model does not capture the yield variability which would be required to identify the 10% lowest yields.

There is no evidence supplied that the difference in yield variability as a consequence of climate variability among the crops can be estimated appropriately, which would be a pre-request to switch one crop against another one to reduce a yield lost in an extreme low yielding year.

These two aspects are fundamental for the conclusions made in this study, which are not supported.

I suggest to reject this paper.

(Remarks on code availability)

Version 1:

Reviewer comments:

Reviewer #1

(Remarks to the Author)
The authors have thoroughly addressed the major comments, and I recommend acceptance. Minor comments:

Line 10: Missing closing parentheses
Line 65-67: Could you add a reference to Fig S3 at the end of this sentence as well?
Line 136: Change "reduce losses" to "reduce drought-related losses" to remind readers that this section does not include all drivers of yield loss.
Line 138: Missing space before (Figure 4) reference.
Line 257: Delete "a" in "and a various other factors..."
Line 357: I think "notice" is supposed to be "note" in this sentence

(Remarks on code availability)

Reviewer #2

(Remarks to the Author)
I co-reviewed this manuscript with one of the reviewers who provided the listed reports. This is part of the Nature Communications initiative to facilitate training in peer review and to provide appropriate recognition for Early Career Researchers who co-review manuscripts.

(Remarks on code availability)

Reviewer #3

(Remarks to the Author)
The Authors have satisfactorily addressed all the concerns raised in the previous review round. More in detail, the authors have revised the description and the statistical comparison by focusing on the distributions of yield anomalies. They have also included additional comparisons with existing model-based estimates of yield anomalies to demonstrate the reliability of the estimates derived from their approach. To assess the consistency of yield responses across the three modeling frameworks, for each crop, the authors compared the distributional properties of the detrended anomalies across models. Pairwise differences among models were evaluated correctly using the two-sample Kolmogorov–Smirnov (KS) test to assess whether the empirical cumulative distributions differ significantly. The requests for additional methodological details have been adequately addressed.

The following are minor revisions to be considered.
-The comment of the results reported in Table S5 (Results of the two-sample KS tests comparing detrended yield anomaly

distributions from the waterCROP model with those from FAOSTAT and LPJmL), as well as the comment of the daily vs monthly resolution ETa comparison are placed inside of the material and methods section. These parts could alternatively be relocated to the Results section.
-The discussion of Table S5 results could further clarify and address that, for potatoes, the KS test comparisons do not indicate good agreement between the waterCROP model estimates and neither of the two benchmarks considered.
-in relation to the KS tests, the author could also point out somewhere (if this has not already been done) that LPJmL includes carbon pools and photosynthetic assimilation processes (in addition to the soil water model), and therefore represents a solid benchmark for comparison.
-lines 395-400 of the trackchange manuscript file: since the test applied KS is standard, the description of its functioning could be summarized
-table 1: the authors could add the reference for the MapSPAM dataset directly in the caption of Table 1 or as a table footnote.
-line 180-183 of the trackchange manuscript file: as previously mentioned, we consider that sustainable irrigation expansion and crop switching should not be referred to as a variety of agricultural interventions; instead, they represent two alternative strategies analyzed in the study. It is possible that the term “variety” remained in the revised text by mistake when referring to these two interventions?

(Remarks on code availability)

Reviewer #4

(Remarks to the Author)
I co-reviewed this manuscript with one of the reviewers who provided the listed reports. This is part of the Nature Communications initiative to facilitate training in peer review and to provide appropriate recognition for Early Career Researchers who co-review manuscripts.

(Remarks on code availability)

Reviewer #5

(Remarks to the Author)
Gridded climate data, spatially explicit agricultural statistics, and empirical water production functions were combined to quantify global patterns of rainfed and irrigated crop-specific drought sensitivity, measured as the percent reduction in median yield under extreme hydroclimatic conditions and drought-associated production losses for 17 major crops, accounting for 75% of global primary crop production. This drought sensitivity metric was used to identify locations where a crop tends to be subject to high climate variability and where crop production may be susceptible to high climate-related production losses. They estimate losses of-10.1% and-6.8% in global rainfed and irrigated median production under historically observed extreme climate conditions and identified hotspots of drought sensitivity in the central US, eastern Brazil, the Mediterranean basin and South Asia. They showed that 62% of production losses for monsoon cereals (rice, maize, millet, sorghum) could be avoided and overall median production been increased by 14% by expanding sustainable irrigation and targeted crop switching.

This is an interesting study and well written. The authors responded already to previous reviewers, have added more information and have clarified and adjusted key definitions. I have no additional comments.

(Remarks on code availability)

Open Access This Peer Review File is licensed under a Creative Commons Attribution 4.0 International License, which permits use, sharing, adaptation, distribution and reproduction in any medium or format, as long as you give appropriate credit to the original author(s) and the source, provide a link to the Creative Commons license, and indicate if changes were made.
In cases where reviewers are anonymous, credit should be given to 'Anonymous Referee' and the source.
The images or other third party material in this Peer Review File are included in the article's Creative Commons license, unless indicated otherwise in a credit line to the material. If material is not included in the article's Creative Commons license and your intended use is not permitted by statutory regulation or exceeds the permitted use, you will need to obtain permission directly from the copyright holder.
To view a copy of this license, visit https://creativecommons.org/licenses/by/4.0/

REVIEWER COMMENTS

Reviewer #1 (Remarks to the Author):

Using historical climate data and process-based crop models, the authors evaluate the climate sensitivity of 17 crops at the grid cell level, which they define as the difference between median yields and yields at the 10th percentile of the evapotranspiration distribution, expressed as a percentage of median yield. They identify hotspots of climate sensitivity with respect to geography and crop type and then evaluate the potential for irrigation expansion and crop switching to reduce climate-related crop losses. This study is well-written, provides new insights into spatial and crop-specific patterns of climate sensitivity, and explores important solutions for improving resilience. The methods for evaluating crop switching benefits are particularly creative and compelling. I have one major concern and a handful of minor concerns that I believe can be addressed with revisions.

Dear Reviewer,

We thank you very much for your thoughtful and constructive review of our manuscript. We are grateful for your positive evaluation of the study's writing quality, the novelty of our findings on spatial and crop-specific patterns of climate sensitivity (drought sensitivity in the revised manuscript), and the creative approach we employed to assess crop switching benefits. We appreciate your recognition of the potential impact of our work and are especially encouraged by your comments on the methodological strengths.

In the following we address your comments (text in italic) point-by-point in blue. In the revised manuscript we have highlighted the changes using the track-changes mode.

Major comments:

The term "climate sensitivity" is used throughout to refer to the percentage reduction in yield that occurs relative to median at the 10th percentile of cumulative seasonal crop ET based on crop model output forced with historical climate data. Based on the calculation of ETa, it seems that this definition of "climate sensitivity" will effectively capture crop stress related moisture-limited conditions, but not necessarily stress related to direct effects of hot or cold temperatures, waterlogging, or shifts in seasonality. The monthly timestep of the climate data further limits the ability of the analysis to capture stress related to short-term moisture limitation and heat stress. Water limited conditions are only responsible for a fraction of total climate-related production losses, and thus, I think that the use of the term "climate sensitivity" and associated phrases like "production losses under extreme climatic conditions" are somewhat overstated and potentially misleading to a cursory reader. This clarification is particularly important in the text discussing the benefits of sustainable irrigation expansion (especially related to Fig 4a), which currently (overoptimistically) implies that irrigation expansion could eliminate 60% of total global climate-related crop losses and >80% of climate-related crop losses in Argentina, Brazil, Nigeria, and the USA. I suggest three revisions related to this issue:

1. The "climate sensitivity" and "extreme climate" terminology should be altered to be more specific, and text should be added to the last paragraph of the introduction and/or the discussion to clarify the types of climate stressors that this analysis can and cannot capture.

We thank the reviewer for this insightful comment. We agree that the term *climate sensitivity* is overly broad relative to the specific phenomena captured by our approach. In the revised manuscript, we now clarify that our indicator refers specifically to yield reductions driven by moisture-limited conditions in the root zone, which lead to decreases in actual crop evapotranspiration (ET_a). Moisture anomalies are derived from the soil water balance computed by the WaterCROP model (developed by the authors), which uses precipitation and reference evapotranspiration inputs over the period 1961–2018. Annual

moisture balances in the root zone are then used to estimate annual ET_a. The resulting pixel-specific (5-arc-minute resolution) ET_a time series is used to quantify crop yield sensitivity to moisture anomalies. Specifically, we evaluate the percentage difference between median yields and the extreme yields associated with the 10th percentile of the ET_a distribution at each pixel. To better reflect the purpose and scope of the indicator, we have introduced the term *crop drought sensitivity*. We also explicitly state in the revised Introduction and Methods sections that our approach does not capture temperature extremes or other forms of climate stress.

In fact, following the reviewer comment, we have better contextualized our index in the known framework of the agricultural drought indicators, aiming at analysing the shortage of water for agriculture, which affects crops yields. So far, various drought indices have been developed to predict short and long term drought conditions such as the Palmer Drought Index (Palmer et al., 1965), Standardized Precipitation Index (McKee et al., 1993), Crop Moisture Index (Palmer et al., 1968), Surface Water Supply Index (Shafer et al., 1982), Vegetation Health Index (Kogan et al., 1995), Vegetation Drought Response Index (Brown et al., 2008). In the table below the key characteristics of these indexes have been shown. We note in particular that all existing drought indices are currently limited to specific regions or require high quality meteorological data.

Table 1 Key indicators to monitor agricultural drought sensitivity.

**Index**	**Typical Basis**	**Strengths**	**Limitations**	**Key References**
SPI (Standardized Precipitation Index)	Precipitation anomalies	Simple, widely used; various timescales	Ignores evaporative demand; not crop-specific	McKee et al. (1993), Proc. AMS Conf. on Applied Climatology
SPEI (Standardized Precipitation-Evapotranspiration Index)	Precipitation minus PET (e.g., Thornthwaite)	Captures both drought duration and intensity; includes temperature	PET method dependent; may not reflect actual crop water stress	Vicente-Serrano et al. (2010), J. Climate
PDSI (Palmer Drought Severity Index)	Soil moisture balance (empirical model)	Long-term drought indicator; widely used in the U.S.	Sensitive to calibration; poor for sub-seasonal or crop-specific analysis	Palmer (1965); Dai (2011), JGR Atmospheres
VCI (Vegetation Condition Index)	NDVI-based vegetation anomaly	Remote sensing of actual plant condition	Reactive, not predictive; may lag drought onset	Kogan (1995), J. Applied Meteorology
DS (Crop-specific Drought sensitivity) (this study)	Actual evapotranspiration (ET_a); 10th percentile; linear yield–water production function	Directly linked to crop stress and yield; combines precipitation and ET demand; high-resolution and crop-specific	Relies on crop parameters (e.g., ky); ET_a modeling dependent	Doorenbos & Kassam (1979), FAO Irrigation and Drainage Paper 33; Steduto et al. (2012), FAO 66; Tuninetti et al. (2015), Water Resources Research

Unlike conventional drought indices that rely solely on meteorological variables, our approach estimates crop-specific yield losses by combining the 10th percentile of actual evapotranspiration (ET_a) with a well-established linear water–yield production function (Doorenbos & Kassam, 1979; Steduto et al., 2012). This method directly captures moisture stress as experienced by crops, integrating both reduced precipitation and elevated atmospheric evaporative demand (i.e., high ET_0) into a single biophysically meaningful metric. While commonly used indices such as the Standardized Precipitation

Index (SPI; McKee et al., 1993), the Standardized Precipitation Evapotranspiration Index (SPEI; Vicente-Serrano et al., 2010), and the Palmer Drought Severity Index (PDSI; Dai, 2011) are valuable for broad-scale drought monitoring, they lack direct links to crop function or productivity. Remote sensing-based indices like the Vegetation Condition Index (VCI; Kogan, 1995) reflect plant responses but tend to lag behind climatic extremes and lack predictive power. In contrast, our ET_a-based index provides a spatially explicit indicator of yield vulnerability grounded in crop water use along the specific growing period, thus offering practical relevance for adaptation planning and resilience assessment.

References

Palmer, W. C. (1965). *Meteorological drought* (Vol. 30). US Department of Commerce, Weather Bureau.

Palmer, W. C. (1968). Keeping track of crop moisture conditions, nationwide: The new crop moisture index.

Shafer, B. A., & Dezman, L. E. (1982). Development of a surface water supply index (SWSI) to assess the severity of drought conditions in snowpack runoff areas.

Kogan, F. N. (1995). Application of vegetation index and brightness temperature for drought detection. *Advances in space research*, *15*(11), 91-100.

Brown, J. F., Wardlow, B. D., Tadesse, T., Hayes, M. J., & Reed, B. C. (2008). The Vegetation Drought Response Index (VegDRI): A new integrated approach for monitoring drought stress in vegetation. *GIScience & Remote Sensing*, *45*(1), 16-46.

McKee, T. B., Doesken, N. J., & Kleist, J. (1993, January). The relationship of drought frequency and duration to time scales. In *Proceedings of the 8th Conference on Applied Climatology* (Vol. 17, No. 22, pp. 179-183).

Vicente-Serrano, S. M., Beguería, S., & López-Moreno, J. I. (2010). A multiscalar drought index sensitive to global warming: the standardized precipitation evapotranspiration index. *Journal of climate*, *23*(7), 1696-1718.

Dai, A. (2011). Characteristics and trends in various forms of the Palmer Drought Severity Index during 1900–2008. *Journal of Geophysical Research: Atmospheres*, *116*(D12).

Kogan, F. N. (1995). Application of vegetation index and brightness temperature for drought detection. *Advances in space research*, *15*(11), 91-100.

2. It would be informative to report the climate conditions under which the 10th percentile ET conditions occur to demonstrate that they are in fact extremes. For example, what are the median temperature and precipitation percentiles associated with the 10th percentile ET? Perhaps the T and P percentiles associated with the 10th percentile of ET could be mapped or the correlations between ET percentiles and climate percentiles plotted to show that low ET is indeed occurring in years with extreme climate conditions.

We thank the reviewer for this comment. We have now included additional analyses and supplementary figures to illustrate the temperature and precipitation conditions under which the 10th percentile ET_a conditions occur, thus demonstrating that they are in fact extreme. We thank the reviewer for this comment because it allowed us to increase the robustness and reliability of our estimates.

For each crop, we determined the year when the 10th percentile ET_a occurred within the study period, and we associated it with the corresponding annual reference evapotranspiration demand (ET_0) and precipitation (P). Then we evaluated if the annual ET_0 and P belong to the low tail of the distribution, thus showing conditions either of higher-than-normal temperature or lower-than-normal rainfall. The results are shown in Figure 1 of this document. For most of the study crops, precipitation is lower than the 20th percentile in >90% of the cultivated pixels, confirming the critical role of rainfall scarcity in inducing moisture anomalies and, thus, yield losses. However, sorghum, millet, rapeseed, and yams exhibit different behaviour as ET_0 anomalies seem to be the most relevant drivers in influencing crop evapotranspiration reduction and, thus, yield. This reflects their relatively high drought tolerance, deep rooting, and capacity to maintain transpiration under moderate water deficits — meaning that typical “dry” years do not push them into the bottom of the 10th ET_a percentile. Instead, their lowest ET_a years occur when atmospheric evaporative demand is reduced, often due to cooler, cloudier, or less windy

conditions that limit energy availability for evapotranspiration. In these cases, the constraint is not water supply (and the soil moisture availability) but reduced climatic drivers of evaporation and transpiration (atmospheric moisture), which can shorten growth duration or limit photosynthesis, ultimately reducing crop water use and productivity. We have inserted this comment in the revised manuscript.

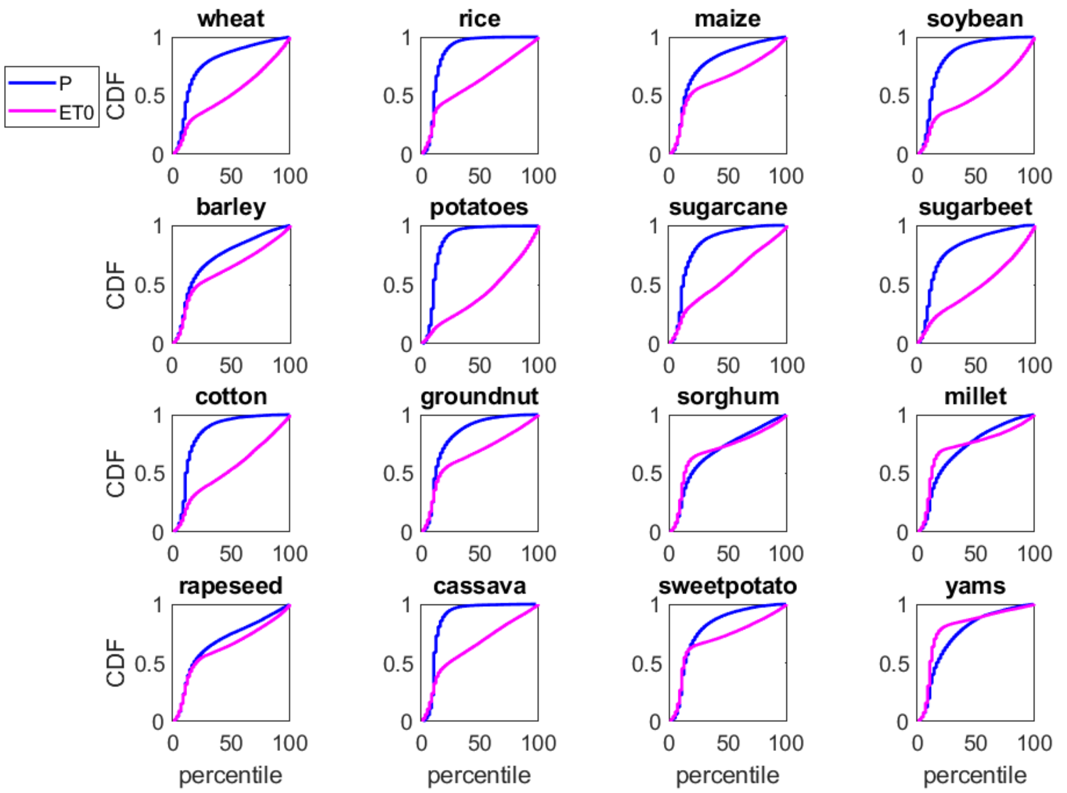

Figure 1 Cumulative Distribution Function (CDF) of rainfall and reference evapotranspiration percentiles assessed in each pixel for the year corresponding to the 10th percentile ETa.

3. Monthly climate data are linearly interpolated to the daily timestep and used as model input. The use of monthly data precludes capturing the effects of shorter-term climate extremes and stressors. I suggest using daily climate data directly, instead of interpolated monthly data.

We thank the reviewer for this valuable comment. While capturing short-term climate extremes and stressors is beyond the scope of this study (though we agree on its relevance), we agree that it is important to assess the robustness of our model. To this end, we performed a validation by comparing model outputs using both monthly and daily input data. Specifically, we used ERA5 data available at both daily and monthly temporal resolutions for rainfall and reference evapotranspiration. The model was run with each dataset to estimate actual evapotranspiration for wheat in the year 2010. The results, presented in Figure 2 below, demonstrate high overall agreement between the two simulations. Under rainfed conditions, we observed slightly higher bias and increased dispersion when using daily versus monthly rainfall data. In irrigated conditions (Figure 2b), there was tight agreement between the two inputs, with a small bias of 7.4 mm over the cropping period. This improved agreement is expected, as rainfall plays a lesser role in irrigated systems where water supplied through irrigation compensates the moisture soil anomaly. Collectively, this demonstrates that our approach captures quite well the overall ET_a outcome and has low sensitivity to the temporal resolution of the input climate data. Thus, in order to save computation time and to limit the data weight, we evaluate the drought sensitivity in this paper using monthly input data. We think that in a future study we could take advantage of the waterCROP model, which now also works with daily input, to better analyse the effects of short term climate extremes along the cropping period and in the different stages of the crop growth.

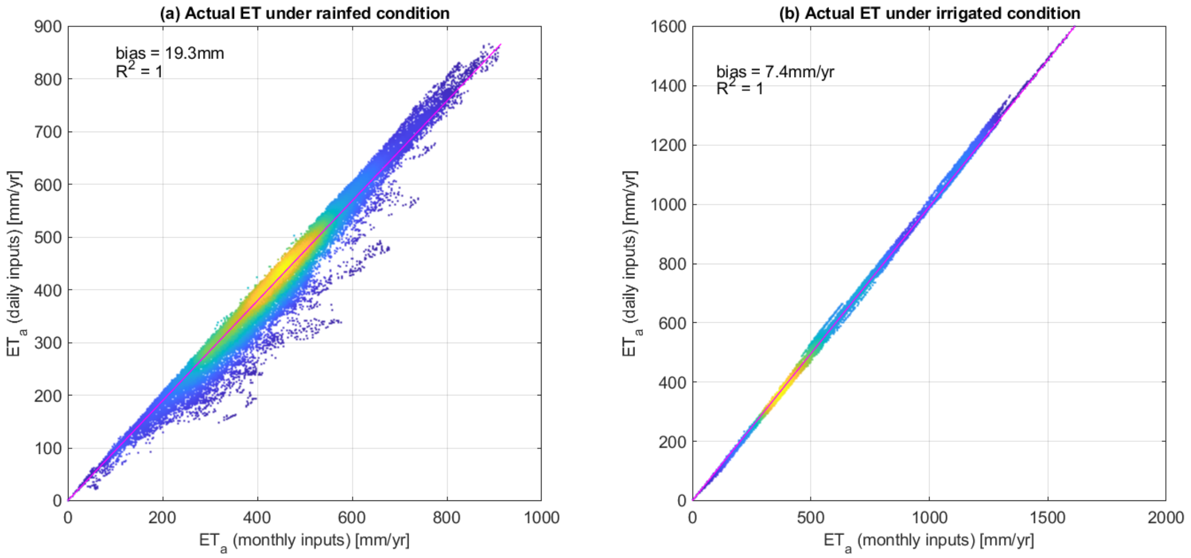

Figure 2 Comparison between the actual crop evapotranspiration obtained for wheat using monthly (x-axis) and daily (y-axis) input data under rainfed (a) and irrigated (b) conditions. The R2 is set to 1 in the panel, but to be more precise it is equal to 0.99995 in panel a and 0.99999 in panel b.

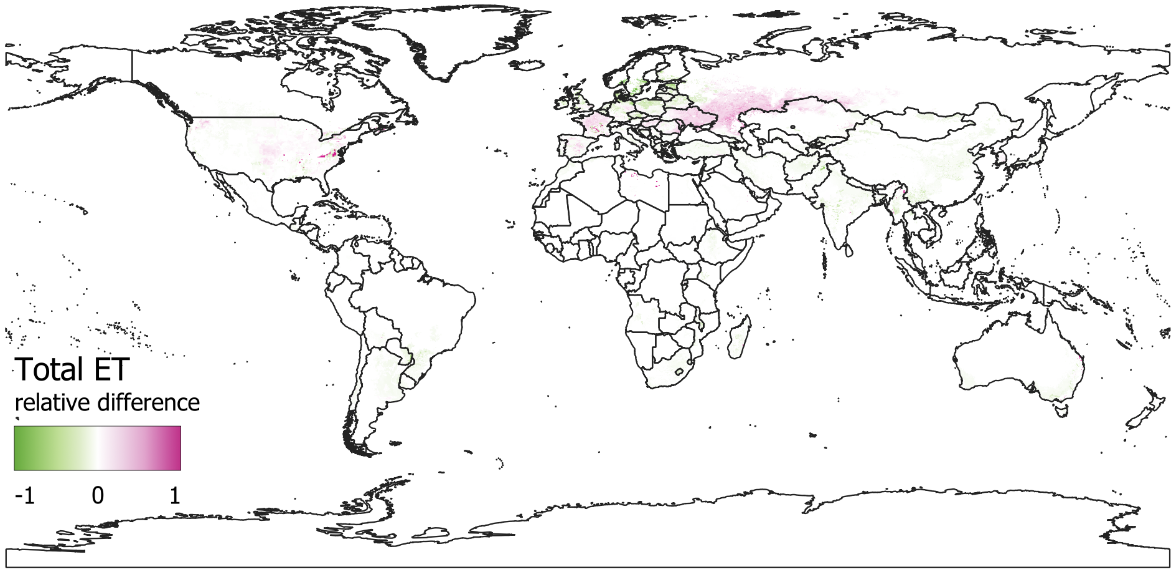

Figure 4 Relative difference between actual wheat evapotranspiration evaluated with daily and monthly input data under rainfed conditions. White pixels show areas where the temporal resolution of the input data does not change the output of the model; green and purple pixels show areas where the resolution of the input data produces smaller or higher changes in the ET estimates.

Minor comments:

Line 23: the phrase "spatially detailed patterns" could be deleted to reduce redundancy with the following line ("at fine spatial granularity").
We have deleted this phrase.

Line 31: Please provide a reference for the statement that these crops constitute 75% of production.

We have now provided a reference.

Line 50-51: Typos, correct "w" to "we" and delete redundant "here" after "of rainfed production".
We have corrected this.

Lines 54-55 (and associated abstract text): These lines state the caloric impact of production losses, but I do not see information in the methods about how production losses were converted to calories. Please add the conversion details to the methods.
Thank you for the comment. In the revised methods section, we have added the details for the conversion of production losses into calorie equivalents.

Line 60: No SI maps are provided for irrigated palm oil or yams; is there no irrigated area for these crops?
According to the dataset we are using there are no irrigated areas for these crops. In the revised manuscript we have added a sentence in the figure's caption to explain it. Thank you for noticing.

Lines 69-71: What is the unit used for rainfed production in these correlations (hectares, tonnes, calories)? Please specify in the SI legend. Also, change "18 crops" to "17 crops".
We have clarified the unit and corrected the number of crops.

Line 76: In addition to not allowing irrigation expansion into areas currently experiencing water scarcity, is the irrigation expansion scenario limited to areas that will be able to support all projected additional irrigation without water scarcity?
We thank the reviewer for this important comment. Indeed, in expanding irrigation in places where water scarcity does not currently exist, it is possible that additional consumptive water demand could induce a transition to conditions of water scarcity in some cases. We had previously highlighted this point within the Discussion section, and we have now added text to make this important consideration more explicit. The revised text reads: "*For irrigation expansion, it will be critical to estimate expected consumptive demand in relation to renewable availability as well as potential knock-on effects including increased competition for water use and land [48], accompanying needs for fertilizer and other inputs [49], impacts on water quality and environmental flows (e.g., [40, 50]), alterations to local climate (e.g., [51]), displacement of smallholders [48], and changes to food access and nutrition [52]. It is also clear that substantial investment would be required in many places in order to enable adequate access and use where water resources are relatively abundant but infrastructure is insufficient (e.g., [36]). In addition, for locations where sustainable irrigation expansion is currently possible to address drought sensitivity hotspots, it will be critical to assess the extent to which climate change may alter the patterns, volume, and timing of water availability in the future. Indeed, the extent to which irrigation can remain a solution for stabilizing and boosting crop production in certain locations depends on several factors including increases in irrigation demand under changing climatic conditions as well as reductions in availability (particularly surface water) through a combination of increased demand and potential alterations in rainfall patterns [39].*".

Line 113: Acronyms for sustainable irrigation expansion and crop switching are introduced here, after the results sections for each of those solutions that did not use acronyms. I recommend omitting the acronyms entirely. If they are retained, introduce them from the first use of each term.
Thank you for the comment. We have omitted the acronyms in the revised manuscript to enhance clarity.

Lines 158-229: I suggest splitting up these very long paragraphs to improve readability. As-written, natural breaks could occur at lines 170, 185, and 215.
Thank you. We have split this text up to improve readability.

Line 141-150: It would be helpful to have some additional context for how the climate sensitivity results from this paper compare with the existing literature on climate impacts on crops in terms of magnitude, spatial patterns, crop-specific differences, etc.

Thank you for this comment. We have added a brief additional text to this section to acknowledge the large amount of past literature on climate impacts and crop production and to state widespread spatial and crop-wise agreement of our estimates with past work. However, given the current length of our manuscript and the many, many studies to which we could compare for selected crops and our quantitative comparisons with gridded crop models and official statistics, we have kept this text relatively short. We have also highlighted some of our most important advances - in disaggregated between irrigated and rainfed production systems and in making some of the first estimates for a large number of understudied crops. The revised text now reads: "Our spatially explicit crop-specific analysis allowed us to identify precise hotspot locations where the drought sensitivity of crops is most pronounced and where actions may be most urgently needed to enhance the climate resilience of crop production. We find that major hotspot regions for rainfed crops exist in the US (soybean, barley, sorghum), India (wheat, soybean, cassava), central Europe (potatoes), and Brazil (potatoes, sugarcane, cassava), among other locations and crops. While a large literature has assessed the impacts of climate on staple crops (e.g., [14, 46, 47]) in agreement with the magnitude and spatial patterns of our estimates, our approach expands this knowledge to a much larger basket of crops (disaggregated between rainfed and irrigated) and at finer spatial resolutions. These advances provide some of the first spatially-detailed estimates of global hotspots for drought sensitivity for a suite of under-studied crops (i.e., those beyond the 'big 4' - maize, rice, soybean, and wheat) and thereby improve the utility of such assessments in supporting climate adaptation interventions. As such, our estimates can also point to locations where economic incentives and agricultural policies may currently be promoting crops that are not well suited agroclimatically - implying that other societal goals are being prioritized at the cost of high crop climate sensitivity".

Line 208: Missing closing parentheses
Thank you. We have added these.

Reviewer #2 (Remarks to the Author):

I co-reviewed this manuscript with one of the reviewers who provided the listed reports. This is part of the Nature Communications initiative to facilitate training in peer review and to provide appropriate recognition for Early Career Researchers who co-review manuscripts.
We thank this reviewer for their positive feedback and insights.

Reviewer #3 (Remarks to the Author):

• *What are the noteworthy results?*

This paper presents an interesting contribution by assessing global crop climate sensitivity and identifying hotspots vulnerable to climate variability. This approach offers insights for enhancing agricultural resilience in climate-sensitive regions, aligning adaptation strategies with food security goals. A new methodology is proposed to evaluate the effect to expand "sustainable irrigation" and evaluating the possibility offered by a crop switching. The work is impressive from the point of view of the data collection and for the ability to manage a very high number of cells, crops and years.
We thank you for recognizing the novelty, scope, and ambition of our analysis. We aimed to develop a scalable framework capable of identifying spatially explicit drought-sensitive hotspots across multiple crops. We appreciate the acknowledgement of our methodological contribution, particularly in combining high-resolution data with novel indicators to support actionable adaptation strategies.

• *Will the work be of significance to the field and related fields? How does it compare to the established literature? If the work is not original, please provide relevant references*

This work is relevant in its attempt to identifying at very detailed scales regions and hotspots where the sensitivity to climate of the crops is hight, and where is possible to increase yield stability using

irrigation or 'sustainable irrigation'. The proposed methodology is new and interesting. This approach expands the scale and detail available in previous literature, that are limited to major crops at country scale. Established literature is considered and the reference are up to date.
We thank the reviewer for this comment. Indeed, much of the existing literature on climate impacts in agriculture operates at coarse spatial scales or focuses on a small set of major crops. Our method advances this field by combining actual evapotranspiration (ET_a)-based stress detection with a yield reduction function at grid-cell and crop-specific levels. We believe this approach adds valuable detail for subnational and regional adaptation planning.

• *Does the work support the conclusions and claims, or is additional evidence needed?*

The work supports its conclusions and claims. However, at least in our opinion, the approach based only on an evaluation of ETa along the year, followed by a set of empirical relations linking ETa to yield losses may be well explained. In this work the soil is only a water reservoir, not a resource that could be compromised (or improved) with different cropping system and relative management. Other important limitation to the productivity as those linked to nutrient availability are not considered. At this detailed scale actually seem very difficult to use cropping system models that consider also other alternative, as nutrient management, tillage option, use of cover crops and so on, but we think that we have to avoid giving the impression that the performance of an agricultural system depends only on the temperature and water. Also, some consideration about extreme events must be done. We think that other cropping system models that include soil dynamics of nutrient, and evolution of organic carbon need to be mentioned. (examples are Daycent, Stics, Armosa, DNDC, APSIM etc…)
We thank the reviewers for their thoughtful comments on the scope and limitations of our modeling framework. We certainly agree that a wide range of biophysical and management factors (such as nutrient availability, tillage practices, organic matter content, and soil degradation) can significantly influence agricultural productivity, and we fully acknowledge that our analysis does not attempt to capture all of these dimensions. Regarding how our model treats soil, we would like to clarify that the soil is not treated merely as a static reservoir in our modeling framework. Instead, key soil properties, such as water holding capacity, are explicitly represented (e.g., IIASA-FAO data at 30 arc second resolution). We also account for dynamic rooting depth over the growing season, which allows the crop to access water from increasing soil volumes as roots develop. This means the model captures spatial and temporal variation in water availability driven by both soil characteristics and plant development. We additionally note that our model's root dynamics also include the consideration of whether the crop is under rainfed or irrigated conditions as this has effects on its ability to reach the deeper soil layer. Notwithstanding, we agree that changes in soil properties due to management practices (e.g., the impact of organic amendments on water retention) could affect moisture availability in the root zone and, thus, crop-specific ET and irrigation needs. While our current study does not address these dynamics directly, we see this as a valuable direction for future work and have added a brief discussion to this effect in the manuscript.
To address the broader comment that our results might overstate the role of water and temperature while omitting other production constraints, we have revised the text in the Introduction, Discussion, and Conclusion to clarify the scope of the analysis. Specifically, we now note that while our results provide insight into moisture-limited yield variability, they do not account for nutrient limitations, pest pressures, or other agronomic factors that also influence yields. We have also included a short discussion of other cropping system models in the Discussion and Conclusion—such as APSIM, DNDC, STICS, and DayCent—that include nutrient cycling and broader soil dynamics, and emphasized the value of integrating such approaches in future work to capture a more holistic picture of climate risk and adaptation in agriculture. Finally, we also added text noting the limitations of our ET-based approach in capturing short-term extreme events (e.g., heatwaves, waterlogging), which can be important drivers of crop loss but are not always reflected in seasonal ET anomalies. We do note however that our estimates remain consistent when utilizing climate data of higher temporal resolution that is able to better represent short-term climate anomalies and extremes.
It is worth mentioning that following this comment and another reviewer's comment, we have also decided to modify the name of our proposed indicator, from climate sensitivity to drought sensitivity. We feel this is a narrower but more accurate term for what we assess in our study. In fact, the indicator

is suited to monitor production losses related to hydroclimatic variabilities and extremes, which impact the soil moisture dynamics and crop–specific evapotranspiration.

• Are there any flaws in the data analysis, interpretation and conclusions? Do these prohibit publication or require revision?

The data analysis and interpretation have some limitations that would benefit from revision to improve the study's rigor but do not prohibit publication. As previously stated, the study claims a process-based approach, but yield modelling relies on empirical functions rather than true process-based yield simulations. The readers have to be conscious of this approach. We think that this potential limitation need to be highlighted, and the text where is supported the quality of this approach comparing statistical data with model result need to be improved.
May be that the recommendations for sustainable irrigation expansion and crop switching are promising but may oversimplify regional variability. For instance (see after) look at the Mediterranean rice's sensitivity, that seem not dependent from water but from critical temperatures during the crop development .
We think that the comparison with already existing model need to be enforced and not limited to the correlation coefficient.
We think that some revision is needed, but the general validity of the work is not under discussion, and there not flaws that prohibit the publication of the manuscript after revision.

We fully agree that agricultural productivity is affected by a broader set of variables, including nutrient dynamics, soil organic matter, and management practices. We have revised the Discussion section to clearly state that our model is limited to water-driven productivity constraints, and does not capture soil nutrient cycling, salinity buildup, or carbon dynamics. We have also added references to relevant process-based models such as APSIM, Daycent, and DNDC, and explained the trade-off between physiological realism and global-scale spatial coverage.

We have further clarified that our approach leverages the FAO yield response to water function—a simplified but robust method well-suited for large-scale sensitivity mapping, and widely adopted for crop water productivity studies (e.g., Doorenbos & Kassam, 1979; Steduto et al., 2012). We agree and have clarified the terminology, changing "process-based" to "empirical ET-yield relationship approach" throughout the manuscript to better reflect our actual methodological framework. We explicitly stated these limitations in the methods and discussion sections, highlighting the focus on moisture limitation while acknowledging other important agricultural factors, including nutrients, carbon, and soil management, which our analysis does not address.

We have also now included additional comparisons with existing model estimates of yield anomalies to demonstrate the reliability of the estimates derived from our approach. "To assess the consistency of yield responses across the three modeling frameworks, we first removed long-term linear trends from each annual yield time series using an ordinary-least-squares de-trending procedure. The resulting anomalies capture interannual variability independent of technological or management improvements. For each crop, we compared the distributional properties of detrended anomalies across models. Pairwise differences among models were evaluated using the two-sample Kolmogorov–Smirnov (KS) test, which examines whether two empirical cumulative distributions differ significantly. The KS test is formulated with the null hypothesis H_0: the two samples are drawn from the same distribution. For each comparison we report the KS statistic together with the test decision variable H, where H = 0 (false) indicates that we cannot reject H_0 and thus the two model anomaly distributions are statistically indistinguishable, whereas H = 1 (true) indicates that we reject H_0, implying that the two models produce significantly different anomaly distributions (see Table below). The test was applied to all pairwise model combinations and repeated across the 16 crops examined. This framework enables us to quantify the degree of agreement among models in representing interannual yield variability while accounting for long-term trends.

Overall, we find strong agreement across the study crops between the anomaly estimates derived from the ET–Y relationship and official FAO yield statistics \cite{fao:stat}: for 12 out of 17 crops, the two sets of anomalies are statistically indistinguishable. A similarly high level of consistency is found when comparing our anomaly estimates with LPJmL outputs for most crops. Some of the differences that we

observe are likely due to the nature of the empirical ET-Y relationships that we leverage, while others may be attributable to relatively poor data quality in official statistics, particularly for lesser-studied crops."

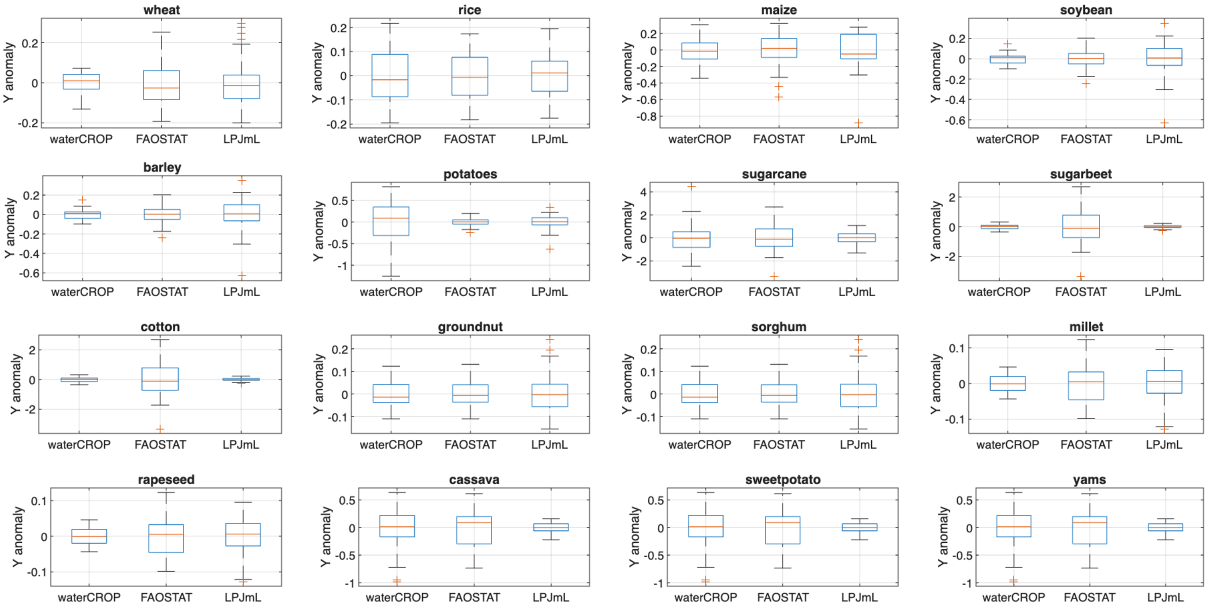

Figure 5. Boxplots of detrended yield anomalies derived from the waterCROP model, FAOSTAT statistics, and the LPJmL model. The anomalies represent interannual yield variability after removal of long-term linear trends. For each crop, the boxplots illustrate the median, interquartile range, and distribution tails of the anomaly series.

Table 1. Results of the two-sample Kolmogorov–Smirnov (KS) tests comparing detrended yield anomaly distributions from the waterCROP model with those from FAOSTAT and LPJmL. For each comparison, the KS statistic, the associated p-value, and the test decision variable H are reported. The null hypothesis (H_0) states that the two samples originate from the same underlying distribution. H = 1 (true) indicates rejection of H_0, implying statistically significant differences between the two anomaly distributions, whereas H = 0 (false) indicates that H_0 cannot be rejected and the distributions are statistically indistinguishable. Higher p-values therefore denote greater similarity between the compared anomaly distributions.

Crop	Models	KSstats	pValue	H
wheat	'waterCROP vs FAOSTAT'	0.3095	0.0277	TRUE
wheat	'waterCROP vs LPJmL'	0.2619	0.0933	FALSE
rice	'waterCROP vs FAOSTAT'	0.0952	0.9874	FALSE
rice	'waterCROP vs LPJmL'	0.1667	0.5642	FALSE
maize	'waterCROP vs FAOSTAT'	0.119	0.9095	FALSE
maize	'waterCROP vs LPJmL'	0.2381	0.1588	FALSE

soybean	'waterCROP vs FAOSTAT'	0.1429	0.752	FALSE
soybean	'waterCROP vs LPJmL'	0.2857	0.0521	FALSE
barley	'waterCROP vs FAOSTAT'	0.1429	0.752	FALSE
barley	'waterCROP vs LPJmL'	0.2857	0.0521	FALSE
potatoes	'waterCROP vs FAOSTAT'	0.4286	5.46E-04	TRUE
potatoes	'waterCROP vs LPJmL'	0.4048	0.0013	TRUE
sugarcane	'waterCROP vs FAOSTAT'	0.0952	0.9874	FALSE
sugarcane	'waterCROP vs LPJmL'	0.2619	0.0933	FALSE
sugarbeet	'waterCROP vs FAOSTAT'	0.381	0.0031	TRUE
sugarbeet	'waterCROP vs LPJmL'	0.2619	0.0933	FALSE
cotton	'waterCROP vs FAOSTAT'	0.381	0.0031	TRUE
cotton	'waterCROP vs LPJmL'	0.2619	0.0933	FALSE
groundnut	'waterCROP vs FAOSTAT'	0.1429	0.752	FALSE
groundnut	'waterCROP vs LPJmL'	0.2381	0.1588	FALSE
sorghum	'waterCROP vs FAOSTAT'	0.1429	0.752	FALSE
sorghum	'waterCROP vs LPJmL'	0.2381	0.1588	FALSE
millet	'waterCROP vs FAOSTAT'	0.2857	0.0521	FALSE
millet	'waterCROP vs LPJmL'	0.2381	0.1588	FALSE
rapeseed	'waterCROP vs FAOSTAT'	0.2857	0.0521	FALSE
rapeseed	'waterCROP vs LPJmL'	0.2381	0.1588	FALSE
cassava	'waterCROP vs FAOSTAT'	0.119	0.9095	FALSE
cassava	'waterCROP vs LPJmL'	0.4048	0.0013	TRUE
sweetpotato	'waterCROP vs FAOSTAT'	0.119	0.9095	FALSE
sweetpotato	'waterCROP vs LPJmL'	0.4048	0.0013	TRUE
yams	'waterCROP vs FAOSTAT'	0.119	0.9095	FALSE

yams	'waterCROP vs LPJmL'	0.4048	0.0013	TRUE

We also thank you for mentioning an example. We agree that in certain regions and for specific crops (like paddy rice in the Mediterranean), water stress may not be the dominant constraint, and that thermal thresholds (e.g., flowering-stage cold spells or heat stress) may play a larger role. We have clarified this in both the Results and Discussion, noting that our framework is most accurate in identifying moisture-related yield losses, and does not capture thermal or phenological stress events. However, utilizing our approach with a more detailed crop model that can incorporate these effects would - in principle - allow such impacts to be captured by our sensitivity estimates. While keeping this in mind, we now explicitly caution against applying our adaptation recommendations without regional agronomic context.

• *Is the methodology sound? Does the work meet the expected standards in your field?*

The methodology is promising and is applied in a clear and reproducible way. There are some minor issue, described in the list of critical point to be addressed.
Thank you for this comment. We have addressed all the issues following the detailed points reported below.

• *Is there enough detail provided in the methods for the work to be reproduced?*

Yes, all the detail to reproduce the methodology and to reproduce the work are adequately described and supported, also with good supplemental material.
Thank you.

Specific comments
Abstract
• *"combining gridded climate data, spatially-explicit agricultural statistics, and process-based crop modeling to quantify global patterns of rainfed and irrigated crop climate sensitivity" in our opinion the term process-based should be substituted.*
Thank you. We have replaced the term "processed-based" with "empirical water-production function" in the abstract.

• *"We estimate -10.1% and -6.8% losses in global rainfed and irrigated production (respectively) under historically observed extreme climate conditions" having used historical data, it is unclear in relation to which productivity values the losses have been calculated*
We thank the reviewer for this question. The production losses are evaluated in the year when the actual evapotranspiration equals the 10th percentile of the distribution with respect to the median management-controlled yield evaluated along the time series from 1961 to 2018. We have specified in the abstract that losses are relative to median management-constant yield.

• *"Our new scalable and universal approach" due to the approach limitations listed in the previous comments, we belive that the term universal should be substituted*
We have removed the term 'universal', and now utilize the phrase "our new scalable and transferable approach".

• *Line 21-23: we belive it would be useful to also mention the application case study, and limitations, of global gridded crop models or the application of process-based model at a national or continental scale in this section (it might be helpful to also move your reference [62] (Müller et al., 2017) here)*
Thank you. We have now included reference to these studies.

Arboleda-Obando, P. F., Ducharne, A., Yin, Z., & Ciais, P. (2024). Validation of a new global irrigation scheme in the land surface model ORCHIDEE v2. 2. Geoscientific Model Development, 17(5), 2141-2164.
Bassu, S., Brisson, N., Durand, J. L., Boote, K., Lizaso, J., Jones, J. W., ... & Waha, K. (2014). How do

various maize crop models vary in their responses to climate change factors?. Global change biology, 20(7), 2301-2320.
Dokoohaki, H., Kivi, M. S., Martinez-Feria, R., Miguez, F. E., & Hoogenboom, G. (2021). A comprehensive uncertainty quantification of large-scale process-based crop modeling frameworks. Environmental Research Letters, 16(8), 084010.
Archontoulis, S. V., Castellano, M. J., Licht, M. A., Nichols, V., Baum, M., Huber, I., ... & Lamkey, K. R. (2020). Predicting crop yields and soil-plant nitrogen dynamics in the US Corn Belt. Crop Science, 60(2), 721-738.
Folberth, C., Elliott, J., Müller, C., Balkovič, J., Chryssanthacopoulos, J., Izaurralde, R. C., ... & Wang, X. (2019). Parameterization-induced uncertainties and impacts of crop management harmonization in a global gridded crop model ensemble. PLoS One, 14(9), e0221862.

• *Line 29-30: Is it possible to indicate the source of the list of crops that make up 75% of global primary production? Wouldn't it be more useful to list these crops in descending order of productivity?*
Thank you for this comment. We evaluated crop-specific production using the MapSPAM dataset (year 2010), which provides both harvested areas and crop yield. Production is the product of these two variables. In table 2 of the main text you can find details about production, including the portion of production under rainfed and irrigated conditions. We prefer to leave this list in alphabetical order, as we believe it will be easier for readers to find any corps of interest.

• *Line 32: Is it possible to provide more detail concerning this daily process-based crop-water model?*
We have now revised this text to state that the model solves a soil water balance on a daily basis for each crop.

• *Line 36: can you elaborate on how the approach employed to estimate the effects of salinity on crop ET and yields (reference [26]) is modified to be integrated in your approach?*
Modeling the effects of soil salinity on crop evapotranspiration and yield lies beyond the scope of this study. We cite Minhas *et al.* at this point in the text because their work provides the most up-to-date estimates of the crop-specific yield response factor (*ky*), which relates anomalies in evapotranspiration to anomalies in yield. We have re-worded this sentence to avoid confusion. While salinity in the root zone can also certainly contribute to yield reductions, this process is not explicitly represented in our analysis. We note, however, that future research could usefully investigate how salinity may exacerbate water stress and affect yield sensitivity.

• *Line 38: It is not very clear why the extreme adverse climate conditions are identified when ETa is below the 10th percentile*
We thank the reviewer for this comment. Low percentile ETa values represent the combination of climatic conditions that would contribute to low productivity for a particular crop. We have also demonstrated (in response to a similar comment by Reviewer #1) that these low ETa values correspond with low percentile precipitation and ET0 years. Regarding the choice of 10th percentile, we feel that this is most appropriate because: a) we are making our assessments over a 60-year time period which is relatively short in order to reliably identify the most extreme ends of a distribution (e.g., 5th percentile or less), and b) this is one of the most commonly used thresholds for a variety of climate assessments and indices (e.g., ETCCDI (Zhang et al., 2011). We have now clarified this point in the manuscript text.

Zhang, X., et al. (2011). "Indices for monitoring changes in extremes based on daily temperature and precipitation data." *Wiley Interdisciplinary Reviews: Climate Change*, 2(6), 851–870.

• *Line 50: Here we focus*
Thank you. We have fixed this.

• *Line 51: "here" is repeated again*
We have corrected this, thank you.

• Line 53: check the brackets
Thank you. We have checked.

• Line 54-55: this sentence, which provides information about irrigated crops in parentheses, is not very smooth to read
We have re-worded this sentence.

• Line 57-58: It should be emphasized that the CS values for these crops confirm information already known from an agronomic point of view
Thank you. We have added a sentence to state this. The added text reads: "*These relative sensitivities are in agreement with agronomic understanding of these crops' drought tolerance (*Daryanto et al. 2017*).*".
Daryanto, Stefani, Lixin Wang, and Pierre-André Jacinthe. "Global synthesis of drought effects on cereal, legume, tuber and root crops production: A review." *Agricultural Water Management* 179 (2017): 18-33.

• Line 80-83: It may be argued that this example is not applicable, particularly in Mediterranean regions. In the Mediterranean Basin, nearly all rice cultivated is paddy rice, with very limited areas irrigated using alternative methods. Rainfed rice does not exist in this context. For rice cultivation, climate sensitivity is primarily attributed to lower temperatures during critical phenological stages of crop development, especially during the flowering phase, rather than the amount of water available.
We have removed this example to avoid confusion.

• Line 115-118: The criteria for crop switching (CrSw) are not written in a very fluid and clear manner. In particular, criterion (i) could be reformulated to enhance clarity
We have reformulated the sentence for clarity and removed the acronyms.

• Line 120: reduce losses by 60%
Thank you. We have revised this wording.

• Line 126-127: Is it possible to elaborate on the reasons?
We have added some brief additional detail.

• Line 146: we belive that SIE and CrSw do not constitute a variety of agricultural intervention, rather, they represent 2 possible strategies that have been analyzed
We agree and we have modified the text accordingly in the revised manuscript.

• Line 170-174: Perhaps a statement regarding the implications of sustainable irrigation expansion on other components of the cropping system (such as nitrogen) should be included, as these aspects are not addressed at all here. This addition would facilitate an acknowledgment and an assessment of the limitations associated with this type of approach
We thank the reviewer for this comment. We have now added text to better highlight these other considerations. The added text reads: "For irrigation expansion, it will be critical to estimate expected consumptive demand in relation to renewable availability as well as potential knock-on effects including increased competition for water use and land [48], accompanying needs for fertilizer and other inputs [49], impacts on water quality and environmental flows (e.g., [40, 50]), alterations to local climate (e.g., [51]), displacement of smallholders [48], and changes to food access and nutrition [52]".

• Line 231-233: This statement should be revised as it is misleading and reflects a reductionist approach. Solar radiation, along with other driving variables affecting agro-ecosystems, plays a crucial role. In particular, radiation is a key variable for estimating ETa.
We thank you for this important comment. We have revised the statement accordingly as ETa depends on reference evapotranspiration that, in turn, depends on radiation, soil heat flux, air temperature, wind speed, and vapor pressure deficit according to the Pennman-Monteith approach.

• *Line 238: Please define the term potential evapotranspiration (PET). The FAO 56 irrigation and drainage paper propose to avoide the use of the term PET due to its potential for varied interpretations. Instead, please use ET_0 to refer to reference evapotranspiration and ETc to refer to crop (maximum) evapotranspiration*
Thank you. We have now included this definition.

• *Line 241-243: Please, show this comparison in some supplemental material.*
Thank you. This comparison is now shown in Figures S1 (Figure 5 below) in the supplementary materials and in Table S5 (Table 1 below), providing the statistics associated with the performed tests.

• *Line 254: Would it be possible to include a brief description of the principles behind the database construction*
We have now added a brief description of the database. The added text reads: "*Global gridded available water content came from the Harmonized World Soil Database (0.5 arcminute, [55]) - a soil information system which integrates regional and national datasets into a consistent framework to provide standardized soil properties and classifications for use in environmental, agricultural, and climate analyses*".

• *Line 260-XX: "We developed a gridded algorithm derived from Tuninetti et al. [22]" even if the algorithm is taken from another publication it could be interesting to shortly describe the alorithm here*
Thank you for the comment. We have added a sentence to explain that the main equation of the waterCROP model is detailed in the Methods text that immediately follows it. We have also changed the word 'algorithm' to 'model'.

• *Line 265: Is root depth variable during phenological stages?*
Yes, the rooting depth varies along the growing period. In particular it typically increases along the first two stages of the cropping period and then, once it has achieved the maximum depth, it remains constant. We have now ensured that we state this in the Methods.

• *Line 267: When the term 'water from precipitation' is used, it may be more accurate to refer to water stored in the soil? This phrasing suggests a direct relationship between rainfall and stress, without acknowledging the mediating role of soil water content*
Thank you. We have re-worded the text accordingly.

• *Equation 2 and 4: Is it necessary to repeat the same equation for calculating CS twice?*
Thank you for this comment. We agree and have removed the second equation in the revised manuscript.

• *Line 288-289 and 295-296: reference format to be fixed*
Thank you. We have done so.

• *Line 298-300: Please provide a brief explanation of why there is a need for conducting a time series correlation analysis. This sentence and the following one is unclear and needs to be reformulated.*
We have revised this description and statistical comparison based on the distributions of yield anomalies. Because our analysis is not dependent on knowing the specific year in which 10th percentile ETa (and its associated yield) occurred, this comparison is the most appropriate and robust for our purposes. We have also now included additional comparisons with existing model estimates of yield anomalies to demonstrate the reliability of the estimates derived from our approach. "To assess the consistency of yield responses across the three modeling frameworks, we first removed long-term linear trends from each annual yield time series using an ordinary-least-squares de-trending procedure. The resulting anomalies capture interannual variability independent of technological or management improvements. For each crop, we compared the distributional properties of detrended anomalies across models. Pairwise differences among models were evaluated using the two-sample Kolmogorov–Smirnov (KS) test, which examines whether two empirical cumulative distributions differ significantly.

The KS test is formulated with the null hypothesis H_0: the two samples are drawn from the same distribution. For each comparison we report the KS statistic together with the test decision variable H, where H = 0 (false) indicates that we cannot reject H_0 and thus the two model anomaly distributions are statistically indistinguishable, whereas H = 1 (true) indicates that we reject H_0, implying that the two models produce significantly different anomaly distributions (see Table below). The test was applied to all pairwise model combinations and repeated across the 16 crops examined. This framework enables us to quantify the degree of agreement among models in representing interannual yield variability while accounting for long-term trends.
Overall, we find strong agreement across the study crops between the anomaly estimates derived from the ET–Y relationship and official FAO yield statistics: for 12 out of 17 crops, the two sets of anomalies are statistically indistinguishable. A similarly high level of consistency is found when comparing our anomaly estimates with LPJmL outputs for most crops. Some of the differences that we observe are likely due to the nature of the empirical ET-Y relationships that we leverage, while others may be attributable to relatively poor data quality in official statistics, particularly for lesser-studied crops."

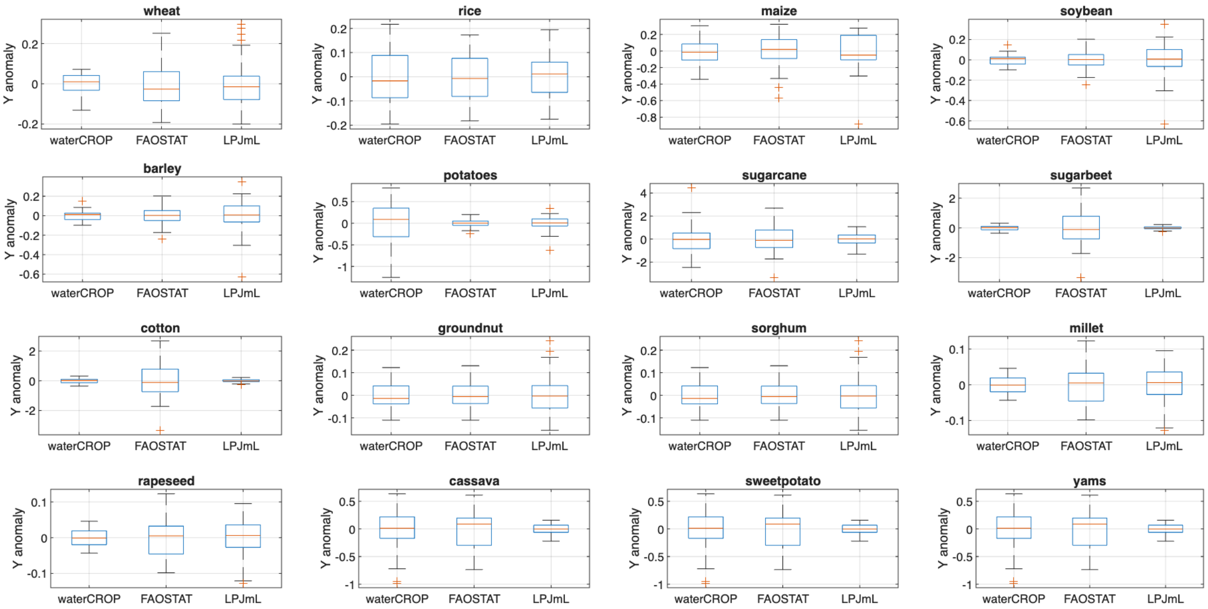

Figure 5. Boxplots of detrended yield anomalies derived from the waterCROP model, FAOSTAT statistics, and the LPJmL model. The anomalies represent interannual yield variability after removal of long-term linear trends. For each crop, the boxplots illustrate the median, interquartile range, and distribution tails of the anomaly series.

Table 1. Results of the two-sample Kolmogorov–Smirnov (KS) tests comparing detrended yield anomaly distributions from the waterCROP model with those from FAOSTAT and LPJmL. For each comparison, the KS statistic, the associated p-value, and the test decision variable H are reported. The null hypothesis (H_0) states that the two samples originate from the same underlying distribution. H = 1 (true) indicates rejection of H_0, implying statistically significant differences between the two anomaly distributions, whereas H = 0 (false) indicates that H_0 cannot be rejected and the distributions are statistically indistinguishable. Higher p-values therefore denote greater similarity between the compared anomaly distributions.

Crop	Models	KSstats	pValue	H
wheat	'waterCROP vs FAOSTAT'	0.3095	0.0277	TRUE

wheat	'waterCROP vs LPJmL'	0.2619	0.0933	FALSE
rice	'waterCROP vs FAOSTAT'	0.0952	0.9874	FALSE
rice	'waterCROP vs LPJmL'	0.1667	0.5642	FALSE
maize	'waterCROP vs FAOSTAT'	0.119	0.9095	FALSE
maize	'waterCROP vs LPJmL'	0.2381	0.1588	FALSE
soybean	'waterCROP vs FAOSTAT'	0.1429	0.752	FALSE
soybean	'waterCROP vs LPJmL'	0.2857	0.0521	FALSE
barley	'waterCROP vs FAOSTAT'	0.1429	0.752	FALSE
barley	'waterCROP vs LPJmL'	0.2857	0.0521	FALSE
potatoes	'waterCROP vs FAOSTAT'	0.4286	5.46E-04	TRUE
potatoes	'waterCROP vs LPJmL'	0.4048	0.0013	TRUE
sugarcane	'waterCROP vs FAOSTAT'	0.0952	0.9874	FALSE
sugarcane	'waterCROP vs LPJmL'	0.2619	0.0933	FALSE
sugarbeet	'waterCROP vs FAOSTAT'	0.381	0.0031	TRUE
sugarbeet	'waterCROP vs LPJmL'	0.2619	0.0933	FALSE
cotton	'waterCROP vs FAOSTAT'	0.381	0.0031	TRUE
cotton	'waterCROP vs LPJmL'	0.2619	0.0933	FALSE
groundnut	'waterCROP vs FAOSTAT'	0.1429	0.752	FALSE
groundnut	'waterCROP vs LPJmL'	0.2381	0.1588	FALSE
sorghum	'waterCROP vs FAOSTAT'	0.1429	0.752	FALSE
sorghum	'waterCROP vs LPJmL'	0.2381	0.1588	FALSE
millet	'waterCROP vs FAOSTAT'	0.2857	0.0521	FALSE
millet	'waterCROP vs LPJmL'	0.2381	0.1588	FALSE
rapeseed	'waterCROP vs FAOSTAT'	0.2857	0.0521	FALSE
rapeseed	'waterCROP vs LPJmL'	0.2381	0.1588	FALSE

cassava	'waterCROP vs FAOSTAT'	0.119	0.9095	FALSE
cassava	'waterCROP vs LPJmL'	0.4048	0.0013	TRUE
sweetpotato	'waterCROP vs FAOSTAT'	0.119	0.9095	FALSE
sweetpotato	'waterCROP vs LPJmL'	0.4048	0.0013	TRUE
yams	'waterCROP vs FAOSTAT'	0.119	0.9095	FALSE
yams	'waterCROP vs LPJmL'	0.4048	0.0013	TRUE

• *Line 300: Please replace "relationship" with correlation*
Thank you. We have done so.
• *Line 301-303: Could you briefly explain in detail why the shift was implemented?*
We have removed this aspect of the analysis, as it is no longer necessary for our comparisons of yield anomaly distributions between data sources.

• *Line 304: It may be worthwhile to comment on the fluctuations of the model for the crops cassava, rice, and sugarcane, as shown in Extended Data Figure 2*
We thank the reviewer for this comment. We note that our estimates are not statistically significantly different from LPJmL for rice or from FAOSTAT for sugarcane. For cassava, the difference is likely attributable to the empirical ETa-Y relationship that we utilize. We have added some brief text to summarize the likely sources of these differences where they infrequently occur.

• *Line 309: Supplementary Figures S38-S42*
Thank you. We have updated this.

• *Line 339: It seems that the Extended Data Figures are not cited in the text according to their numbering, so it may be necessary to rearrange them*
Thank you for pointing this out, and we apologize for this oversight. We have now ensured that we reference the Extended Data Figures in the main text.

• *Figure 1: It may be advisable to reconsider the font sizes on the horizontal axes labels of the bar plots dedicated to the countries. Additionally, it is unclear how these 6 countries featured in the bar plots were selected, can you elaborate in the figure caption?*
Thank you. We have ensured that the font size is legible on the horizontal axes of the inset plots. We have revised the figure to enhance its readability and improve clarity. We have decided to only put two significant examples, Brazil and China, which were selected because they provide geographic diversity and cultivated crops showing different sensitivity. We have added this explanation in the figure caption. We have added the country-scale results for all countries and crops in Tables S2 and S3 of the revised manuscript.

Reviewer #4 (Remarks to the Author):

I co-reviewed this manuscript with one of the reviewers who provided the listed reports. This is part of the Nature Communications initiative to facilitate training in peer review and to provide appropriate recognition for Early Career Researchers who co-review manuscripts.
We thank this reviewer for their positive feedback and insights.

Reviewer #5 (Remarks to the Author):

Gridded climate data, spatially-explicit agricultural, statistics, and the Watercrop model based on a simple yield-ET function, were combined to quantify global patterns of rainfed and irrigated crop climate sensitivity and climate-associated production losses for 17 major crops. A -10.1% and -6.8% losses in global rainfed and irrigated production was estimated under historically observed extreme climate conditions with hotspots of climate sensitivity in the central US, eastern Brazil, the Mediterranean basin, and South Asia. To illustrate how sustainable irrigation expansion and targeted crop switching could reduce climate sensitivity, a case was introduced for rice, maize, millet, sorghum replacing each other and it was shown that 62% of production losses could be avoided while increasing overall production by 14%. It was concluded that this approach in measuring climate sensitivity of crops enables the assessment of where climate-related production losses tend to be largest and where mitigating actions and investments can be proactively targeted to better ensure the stability and increased supply of global crop production.
While this is an interesting idea of exploring the potential of irrigation where water is sustainable availability and crop switching to reduce the impact of season-to-season variability of low yields on global food production, the applied Watercrop model seems an unsuitable tool this task. The validation of comparing the Watercrop model with another benchmark model and FAO yield data shows mostly no correlation and in some cases high yields when the other model or FAO yield data show low yields and visa versa. The Watercrop model does not capture the yield variability which would be required to identify the 10% lowest yields.
There is no evidence supplied that the difference in yield variability as a consequence of climate variability among the crops can be estimated appropriately, which would be a pre-request to switch one crop against another one to reduce a yield lost in an extreme low yielding year.
These two aspects are fundamental for the conclusions made in this study, which are not supported.
I suggest to reject this paper.
We sincerely thank the reviewer for their interest in our paper and for raising this important methodological point - which centers on the reviewer's view that our model does not sufficiently capture yield variability. We respectfully disagree with the conclusion that the *WaterCROP* model is unsuitable for this analysis and have substantially expanded the manuscript to clarify and demonstrate its robustness and applicability for the stated objectives. We apologize for the confusion that our previous time series comparisons caused and have revised our validations for ease of understanding and to clearly demonstrate the effectiveness of our approach is reliably replicating interannual yield variability. Because our analysis is not dependent on knowing the specific year in which 10th percentile ETa (and its associated yield) occurred, this comparison is the most appropriate and robust for our purposes. We have also now included additional comparisons with existing model estimates of yield anomalies to demonstrate the reliability of the estimates derived from our approach.
Specifically, we have now performed a suite of statistical comparisons with official statistics from FAOSTAT and global gridded crop model estimates from the globally recognized LPJmL model. We have also now included additional comparisons with existing model estimates of yield anomalies to demonstrate the reliability of the estimates derived from our approach. "To assess the consistency of yield responses across the three modeling frameworks, we first removed long-term linear trends from each annual yield time series using an ordinary-least-squares de-trending procedure. The resulting anomalies capture interannual variability independent of technological or management improvements. For each crop, we compared the distributional properties of detrended anomalies across models. Pairwise differences among models were evaluated using the two-sample Kolmogorov–Smirnov (KS) test, which examines whether two empirical cumulative distributions differ significantly. The KS test

is formulated with the null hypothesis H_0: the two samples are drawn from the same distribution. For each comparison we report the KS statistic together with the test decision variable H, where H = 0 (false) indicates that we cannot reject H_0 and thus the two model anomaly distributions are statistically indistinguishable, whereas H = 1 (true) indicates that we reject H_0, implying that the two models produce significantly different anomaly distributions (see Table below). The test was applied to all pairwise model combinations and repeated across the 16 crops examined. This framework enables us to quantify the degree of agreement among models in representing interannual yield variability while accounting for long-term trends.

Overall, we find strong agreement across the study crops between the anomaly estimates derived from the ET–Y relationship and official FAO yield statistics: for 12 out of 17 crops, the two sets of anomalies are statistically indistinguishable. A similarly high level of consistency is found when comparing our anomaly estimates with LPJmL outputs for most crops. Some of the differences that we observe are likely due to the nature of the empirical ET-Y relationships that we leverage, while others may be attributable to relatively poor data quality in official statistics, particularly for lesser-studied crops."

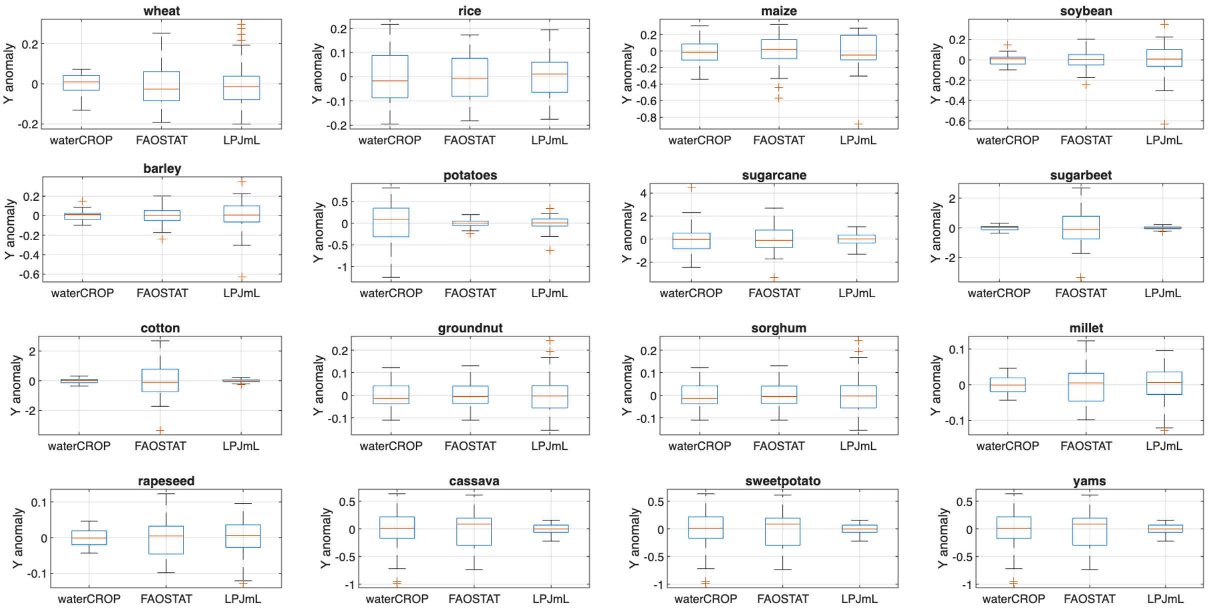

Figure 5. Boxplots of detrended yield anomalies derived from the waterCROP model, FAOSTAT statistics, and the LPJmL model. The anomalies represent interannual yield variability after removal of long-term linear trends. For each crop, the boxplots illustrate the median, interquartile range, and distribution tails of the anomaly series.

Table 1. Results of the two-sample Kolmogorov–Smirnov (KS) tests comparing detrended yield anomaly distributions from the waterCROP model with those from FAOSTAT and LPJmL. For each comparison, the KS statistic, the associated p-value, and the test decision variable H are reported. The null hypothesis (H_0) states that the two samples originate from the same underlying distribution. H = 1 (true) indicates rejection of H_0, implying statistically significant differences between the two anomaly distributions, whereas H = 0 (false) indicates that H_0 cannot be rejected and the distributions are statistically indistinguishable. Higher p-values therefore denote greater similarity between the compared anomaly distributions.

**Crop**	**Models**	**KSstats**	**pValue**	**H**
wheat	'waterCROP vs FAOSTAT'	0.3095	0.0277	TRUE

wheat	'waterCROP vs LPJmL'	0.2619	0.0933	FALSE
rice	'waterCROP vs FAOSTAT'	0.0952	0.9874	FALSE
rice	'waterCROP vs LPJmL'	0.1667	0.5642	FALSE
maize	'waterCROP vs FAOSTAT'	0.119	0.9095	FALSE
maize	'waterCROP vs LPJmL'	0.2381	0.1588	FALSE
soybean	'waterCROP vs FAOSTAT'	0.1429	0.752	FALSE
soybean	'waterCROP vs LPJmL'	0.2857	0.0521	FALSE
barley	'waterCROP vs FAOSTAT'	0.1429	0.752	FALSE
barley	'waterCROP vs LPJmL'	0.2857	0.0521	FALSE
potatoes	'waterCROP vs FAOSTAT'	0.4286	5.46E-04	TRUE
potatoes	'waterCROP vs LPJmL'	0.4048	0.0013	TRUE
sugarcane	'waterCROP vs FAOSTAT'	0.0952	0.9874	FALSE
sugarcane	'waterCROP vs LPJmL'	0.2619	0.0933	FALSE
sugarbeet	'waterCROP vs FAOSTAT'	0.381	0.0031	TRUE
sugarbeet	'waterCROP vs LPJmL'	0.2619	0.0933	FALSE
cotton	'waterCROP vs FAOSTAT'	0.381	0.0031	TRUE
cotton	'waterCROP vs LPJmL'	0.2619	0.0933	FALSE
groundnut	'waterCROP vs FAOSTAT'	0.1429	0.752	FALSE
groundnut	'waterCROP vs LPJmL'	0.2381	0.1588	FALSE
sorghum	'waterCROP vs FAOSTAT'	0.1429	0.752	FALSE
sorghum	'waterCROP vs LPJmL'	0.2381	0.1588	FALSE
millet	'waterCROP vs FAOSTAT'	0.2857	0.0521	FALSE
millet	'waterCROP vs LPJmL'	0.2381	0.1588	FALSE
rapeseed	'waterCROP vs FAOSTAT'	0.2857	0.0521	FALSE
rapeseed	'waterCROP vs LPJmL'	0.2381	0.1588	FALSE

cassava	'waterCROP vs FAOSTAT'	0.119	0.9095	FALSE
cassava	'waterCROP vs LPJmL'	0.4048	0.0013	TRUE
sweetpotato	'waterCROP vs FAOSTAT'	0.119	0.9095	FALSE
sweetpotato	'waterCROP vs LPJmL'	0.4048	0.0013	TRUE
yams	'waterCROP vs FAOSTAT'	0.119	0.9095	FALSE
yams	'waterCROP vs LPJmL'	0.4048	0.0013	TRUE

Together, these validations convincingly demonstrate that our model performs comparable - and in some cases superior - to state-of-the-art global gridded crop models and is able to accurately capture interannual yield variability for most crops in comparison to official statistics. As such, we feel that all of our comprehensive revisions, additional validations, and clarifications address the reviewer's point and demonstrate the robustness of our approach and conclusions.

REVIEWER COMMENTS

Reviewer #1 (Remarks to the Author):

The authors have thoroughly addressed the major comments, and I recommend acceptance. Minor comments:

Many thanks for all your inputs and comments to this paper. We have now addressed all the remaining minor comments.

Line 10: Missing closing parentheses

Done, thank you.

Line 65-67: Could you add a reference to Fig S3 at the end of this sentence as well?

Done, thank you.

Line 136: Change "reduce losses" to "reduce drought-related losses" to remind readers that this section does not include all drivers of yield loss.

Done, thank you.

Line 138: Missing space before (Figure 4) reference.

Done, thank you.

Line 257: Delete "a" in "and a various other factors..."

Done, thank you.

Line 357: I think "notice" is supposed to be "note" in this sentence

Done, thank you.

Reviewer #2 (Remarks to the Author):

I co-reviewed this manuscript with one of the reviewers who provided the listed reports. This is part of the Nature Communications initiative to facilitate training in peer review and to provide appropriate recognition for Early Career Researchers who co-review manuscripts.

Thank you for your constructive feedback and input throughout the review process.

Reviewer #3 (Remarks to the Author):

The Authors have satisfactorily addressed all the concerns raised in the previous review round. More in detail, the authors have revised the description and the statistical comparison by focusing on the distributions of yield anomalies. They have also included additional comparisons with existing model-based estimates of yield anomalies to demonstrate the reliability of the estimates derived from their approach. To assess the consistency of yield responses across the three modeling frameworks, for

each crop, the authors compared the distributional properties of the detrended anomalies across models. Pairwise differences among models were evaluated correctly using the two-sample Kolmogorov–Smirnov (KS) test to assess whether the empirical cumulative distributions differ significantly. The requests for additional methodological details have been adequately addressed.

Many thanks for all your inputs and comments to this paper. We have now addressed all the remaining minor comments.

The following are minor revisions to be considered.
-The comment of the results reported in Table S5 (Results of the two-sample KS tests comparing detrended yield anomaly distributions from the waterCROP model with those from FAOSTAT and LPJmL), as well as the comment of the daily vs monthly resolution ETa comparison are placed inside of the material and methods section. These parts could alternatively be relocated to the Results section.

Thank you. In order to maintain the flow of the Results text, we prefer to keep the KS tests and daily vs monthly ETa comparisons in their current location.

-The discussion of Table S5 results could further clarify and address that, for potatoes, the KS test comparisons do not indicate good agreement between the waterCROP model estimates and neither of the two benchmarks considered.

We have added text to state this, thank you. We have also added references supporting our findings showing a non-negligible impacts of droughts on potatoes productivities.

-in relation to the KS tests, the author could also point out somewhere (if this has not already been done) that LPJmL includes carbon pools and photosynthetic assimilation processes (in addition to the soil water model), and therefore represents a solid benchmark for comparison.

Done, thank you.

-lines 395-400 of the trackchange manuscript file: since the test applied KS is standard, the description of its functioning could be summarized

Done, thank you.

-table 1: the authors could add the reference for the MapSPAM dataset directly in the caption of Table 1 or as a table footnote.

Done, thank you.

-line 180-183 of the trackchange manuscript file: as previously mentioned, we consider that sustainable irrigation expansion and crop switching should not be referred to as a variety of agricultural interventions; instead, they represent two alternative strategies analyzed in the study. It is possible that the term "variety" remained in the revised text by mistake when referring to these two interventions?

Thank you, we have replaced this with "alternative agricultural strategies"

Reviewer #4 (Remarks to the Author):

I co-reviewed this manuscript with one of the reviewers who provided the listed reports. This is part of the Nature Communications initiative to facilitate training in peer review and to provide appropriate recognition for Early Career Researchers who co-review manuscripts.

Thank you for your constructive feedback and input throughout the review process.

Reviewer #5 (Remarks to the Author):

Gridded climate data, spatially explicit agricultural statistics, and empirical water production functions were combined to quantify global patterns of rainfed and irrigated crop-specific drought sensitivity, measured as the percent reduction in median yield under extreme hydroclimatic conditions and drought-associated production losses for 17 major crops, accounting for 75% of global primary crop production. This drought sensitivity metric was used to identify locations where a crop tends to be subject to high climate variability and where crop production may be susceptible to high climate-related production losses. They estimate losses of-10.1% and-6.8% in global rainfed and irrigated median production under historically observed extreme climate conditions and identified hotspots of drought sensitivity in the central US, eastern Brazil, the Mediterranean basin and South Asia. They showed that 62% of production losses for monsoon cereals (rice, maize, millet, sorghum) could be avoided and overall median production been increased by 14% by expanding sustainable irrigation and targeted crop switching.

This is an interesting study and well written. The authors responded already to previous reviewers, have added more information and have clarified and adjusted key definitions. I have no additional comments.

Thank you for your constructive feedback and input throughout the review process.
